# Fluorescent Liquid Tetrazines

**DOI:** 10.3390/molecules26196047

**Published:** 2021-10-06

**Authors:** Maximilian Paradiz Dominguez, Begüm Demirkurt, Marion Grzelka, Daniel Bonn, Laurent Galmiche, Pierre Audebert, Albert M. Brouwer

**Affiliations:** 1 Van’t Hoff Institute for Molecular Sciences, Faculty of Science, University of Amsterdam, Science Park 904, 1098 XH Amsterdam, The Netherlands; m.paradizdominguez@uva.nl (M.P.D.); b.demirkurt@uva.nl (B.D.); 2 Institute of Physics, Faculty of Science, University of Amsterdam, Science Park 904, 1098 XH Amsterdam, The Netherlands; m.grzelka@uva.nl (M.G.); d.bonn@uva.nl (D.B.); 3 PPSM, ENS Cachan, CNRS, Université Paris Saclay, 94235 Cachan, France; Laurent.GALMICHE@ppsm.ens-cachan.fr (L.G.); audebert@ppsm.ens-cachan.fr (P.A.); 4 Advanced Research Center for Nanolithography, Science Park 106, 1098 XG Amsterdam, The Netherlands

**Keywords:** fluorescence, rheology, viscosity, excited states, inter system crossing

## Abstract

Tetrazines with branched alkoxy substituents are liquids at ambient temperature that despite the high chromophore density retain the bright orange fluorescence that is characteristic of this exceptional fluorophore. Here, we study the photophysical properties of a series of alkoxy-tetrazines in solution and as neat liquids. We also correlate the size of the alkoxy substituents with the viscosity of the liquids. We show using time-resolved spectroscopy that intersystem crossing is an important decay pathway competing with fluorescence, and that its rate is higher for 3,6-dialkoxy derivatives than for 3-chloro-6-alkoxytetrazines, explaining the higher fluorescence quantum yields for the latter. Quantum chemical calculations suggest that the difference in rate is due to the activation energy required to distort the tetrazine core such that the $n\pi^{*}$$S_{1}$ and the higher-lying $\pi \pi^{*}$$T_{2}$ states cross, at which point the spin-orbit coupling exceeding 10 cm${}^{-1}$ allows for efficient intersystem crossing to occur. Femtosecond time-resolved anisotropy studies in solution allow us to measure a positive relationship between the alkoxy chain lengths and their rotational correlation times, and studies in the neat liquids show a fast decay of the anisotropy consistent with fast exciton migration in the neat liquid films.

## 1. Introduction

Molecular materials that are liquid under ambient conditions and show luminescence in the visible spectral range have drawn increasing attention during the past decade [1,2,3]. Such materials are somewhat uncommon because chromophores with a π-system that is sufficiently extended to give visible fluorescence tend to have strong intermolecular interactions that cause the pure material to solidify. Moreover, allowing the chromophores to interact via π-stacking often leads to the loss of fluorescence, e.g., in H-aggregates [4]. The use of branched alkyl groups as side chains is a well established design principle to prevent inter-chromophore interaction and crystallization, which has allowed liquefaction of a number of chromophores of moderate size [3]. Another elegant strategy is to exploit the entropy of mixing of two compounds with the same chromophore and slightly different substituent groups [5,6].

The fluorescence of most organic molecules at high concentrations or as neat materials is strongly influenced by intermolecular interactions between neighboring fluorophores, leading to changes in the spectra, lifetimes, and quantum yields. In concentrated solutions, well-defined aggregates or excimers may be formed, which typically give red-shifted emission compared to the non-interacting chromophore [7]. When the luminescence intensity is increased due to aggregation, we speak of aggregation-induced emission, a phenomenon that has been studied extensively in the past two decades [8,9].

Making use of the smallest stable visible chromophore, 1,2,4,5-tetrazine, Allain et al. [10] succeeded in making a fluorescent liquid with a relatively low viscosity and fluorescence spectra and quantum yields that are almost unchanged relative to those of the same compound in solution [11]. Subsequently, these compounds were applied in quantitative imaging of the contact between objects [12] and in a study of lubrication [13].

The fluorescence efficiency of tetrazines depends on their substitution at the 3- and 6-positions. The parent system emits very weakly, [14] to a large extent due to photodecomposition [15,16]. 3,6-Substituted derivatives are photostable, and some show strong fluorescence, including the dialkoxy and chloroalkoxy derivatives [10,17,18]. Other derivatives, e.g., with aromatic substituents, on the other hand, emit only weakly [19,20]. A remarkable feature of the tetrazine fluorescent liquids is that the absorption and luminescence spectra of the chromophore are virtually unchanged in the neat liquid state. In larger π-systems such as liquefied versions of naphthalene, carbazole, fluorene, pyrene, and others, the chromophores in the neat liquid do still interact sufficiently to give relatively large Stokes shifts [2,3]. The electron deficient tetrazine rings, on the other hand, have little or no tendency towards π-stacking [21]. Moreover, the lowest-energy transition has a small transition dipole moment, leading to an intrinsically small coupling with neighboring molecules [22].

**Scheme 1:**
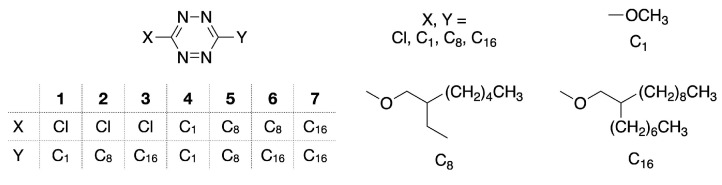
Compounds studied.

The s-tetrazine derivatives with branched alkoxy substituents have rather low viscosities. In the context of our studies of lubrication, we were interested in increasing the viscosity of the fluorescent liquids by increasing the size of the alkoxy groups. As a side effect, this increases the average distance between neighboring chromophores, which may have an effect on the fluorescence quantum yield. The length of the chains has no effect on the photophysical properties of the molecules in solution. However, the fluorescence lifetime is considerably longer for $ClC_{16}$ than $ClC_{8}$ as a neat liquid, and longer chains do lead to a very small increase of the fluorescence lifetime of the dialkoxy compounds (Table S11). By analyzing the time-resolved dynamics of members of this class of molecules (Scheme 1), we have determined some of their most important photophysical properties. We show that intersystem crossing to the triplet state is the main non-radiative decay pathway of the fluorescent liquids when dissolved in dichloromethane (DCM). We conclude, on the basis of quantum chemical calculations, that the rate of intersystem crossing depends on the activation energy required to distort the tetrazine core to a geometry at which the $S_{1}$ ($n\pi^{*}$) and the higher-lying $T_{2}$ ($\pi \pi^{*}$) states become degenerate, and that this activation energy is lower for the dialkoxy series than for the chloroalkoxy. Rheological measurements on small quantities of the liquids have allowed us to determine how the length of the alkoxy chains affects the viscosity of the liquids, and time-resolved anisotropy in solution reveals how these chains slow down the rotation of the molecule in solution in a way consistent with the Einstein–Stokes model of rotational diffusion. Finally, we show that the anisotropy decays much faster in the neat liquid than would be expected from pure rotational diffusion in a viscous media, and we explain this observation on the basis of exciton diffusion between neighboring molecules.

## 2. Materials and Methods

### 2.1. Synthesis and Characterization

All reagents and solvents were purchased from commercial sources (Aldrich, Acros, SDS) and used as received. Spectroscopic grade solvents purchased from Aldrich were used for spectroscopic measurements. Analytical TLC was performed on Kieselgel F-254 precoated plates. Visualization was done with a UV lamp. Flash chromatography was carried out with silica gel 60 (230–400 mesh) from SDS. ${}^{1}H$ and ${}^{13}C$ NMR spectra were recorded on a JEOL 400 MHz spectrometer, and chemical shifts (δ) are reported in ppm relative to TMS and referenced to the residual solvent. 3,6-Dichloro-s-tetrazine was prepared according to a literature procedure [10,23].

*3-Chloro-6-((2-hexyldecan-1-yl)oxy)-1,2,4,5-tetrazine* (${\mathrm{ClC}}_{16}$). 2,4,6-Collidine (529 μL, 485 mg, 4.00 mmol, 1.0 eq.) is added to a solution of 3,6-dichlorotetrazine (604 mg, 4.00 mmol, 1.0 eq.) and 2-hexyl-1-decanol (1.0 g, 4.15 mmol, 1.0 eq) in dry dichloromethane (80 mL). The resulting solution is stirred for 4 h, then concentrated and purified by column chromatography (eluent petroleum ether/dichloromethane 7/3) to yield **$ClC_{16}$** as a pink fluorescent liquid (742 mg, 70% yield). ${}^{1}H$ NMR 4.56, d (2H, $O-C{\underline{H}}_{2}-CH$), 2.00–1.91 (1H, m $CH_{2}-C\underline{H}\left(RR^{\prime }\right)$), 1.55–1.25 (24H, not resolved, chain alkyls) 0.93-0.88 (6H, not resolved, chain ends). See Figure S1. ${}^{13}C$ NMR 166.89 ($tetrazine-OC_{16}$), 164.12 (Cl−tetrazine), 73.73 ($O-\underline{C}H_{2}$), 37.53, 31.87, 31.79, 30.96, 29.89, 29.56, 29.29, 26.71, 22.67, 14.11 (alkyls). See Figure S2.

*3,6-bis-((2-hexyldecan-1-yl)oxy)-1,2,4,5-tetrazine ($C_{16}C_{16}$*). 2,4,6-Collidine (529 μL, 485 mg, 4.00 mmol, 1.0 eq.) is added to a solution of 3,6-dichlorotetrazine (604 mg, 4.00 mmol, 1.0 eq.) and 2-hexyl-1-decanol (2.2 g, 9 mmol, 2.25 eq) in dichloromethane (80 mL). The resulting solution is stirred for 5 h. Then, 4-dimethylaminopyridine (1.074 g, 8.80 mmol, 2.2 eq) is added in one portion. The reaction mixture is stirred overnight then concentrated and purified by column chromatography (eluent petroleum ether/dichloromethane from 8/2 to 7/3) to yield **$C_{16}C_{16}$** as a pink fluorescent liquid (1.0 g, 40% yield). ${}^{1}H$ NMR 4.43, d (2H, $O-C{\underline{H}}_{2}-CH$) 1.91 (1H, m $CH_{2}-C\underline{H}\left(RR^{\prime }\right)$), 1.34–1.27 (24H, not resolved, chain alkyls) 0.89-0.86 (6H, not resolved, chain ends). See Figure S3. ${}^{13}C$ NMR 166.41 (tetrazine) 72.48 ($O-\underline{C}H_{2}$) 37.76, 32.03, 31.98, 31.20, 30.97, 29.73, 29.46, 26.88, 22.81, 14.24 (alkyls). See Figure S4.

*3-((2-ethyl-1-hexyl)oxy)-6-((2-hexyldecan-1-yl)oxy)-1,2,4,5-tetrazine* ($C_{8}C_{16}$). 2,4,6-Collidine (529 μL, 485 mg, 4.00 mmol, 1.0 eq.) is added to a solution of 3,6-dichlorotetrazine (604 mg, 4.00 mmol, 1.0 eq.) and 2-hexyl-1-decanol (1.0 g, 4.15 mmol, 1.0 eq) in dichloromethane (80 mL). The resulting solution is stirred for 5 h. After this time, 0.5 g of 2-ethyl-1-hexanol and 4-dimethylaminopyridine (1.074 g, 8.80 mmol, 2.2 eq) are added in one portion. The reaction mixture is stirred for an additional 2 h, then concentrated and purified by column chromatography (eluent petroleum ether/dichloromethane from 10/1 to 7/3) to yield $C_{8}C_{16}$ as a pink fluorescent liquid (1.0 g, 25% yield, second and main product eluted). The same column allows to separate 3,6-bis-(2-ethylhex-1-yl)-tetrazine $C_{8}C_{8}$ (first eluted, 10%) and 3, 6-bis-(2-hexyldecan-1-yl)-tetrazine $C_{16}C_{16}$ (last eluted, 5%) as side products. ${}^{1}H$ NMR 4.46-4.41, m (4H, $O-C{\underline{H}}_{2}-CH$) 1.89–1.85 (2H, m $CH_{2}-C\underline{H}\left(RR^{\prime }\right)$), 1.34–1.27 (32H, not resolved, chain alkyls) 0.96–0.86 (12H, not resolved, chain ends). See Figure S5. ${}^{13}C$ NMR 166.40 (tetrazine) 72.81, 72.42 ($O-\underline{C}H_{2}$) 39.14, 39.91, 32.03, 31.97, 31.19, 30.37, 29.75, 29.71, 29.45, 29.11, 26.88, 26.86, 23.75, 23.13, 22.80, 14.23, 14.19, 11.14 (alkyls). See Figure S6.

### 2.2. Rheometry

Rheology measurements were performed on an Anton Paar MCR302 rheometer using a cone-plate geometry (diameter 12mm, angle 1${}^{\circ }$). This geometry is chosen to use a small amount of liquid (∼10 μL). The Newtonian viscosities of four different fluorescent liquids were measured by steady shear at T=20°C and over a shear rate range of [10–100]$\;s^{-1}$. Before measurement, all fluorescent liquids were pumped under vacuum by turbo pump (∼30 min) to remove any traces of organic solvents that might have remained from the synthesis.

### 2.3. Steady State Spectra

UV/Vis absorption spectra were recorded using a Shimadzu UV2700 spectrometer. Fluorescence emission spectra were measured using a Spex 3 Fluorolog. For quantum yields, a standard protocol for relative quantum yield measurements was followed, using Rhodamine 101 in ethanol as a reference ($\phi_{f}=91.5\%$) [24], and an excitation wavelength of 525 nm.

### 2.4. Time-Resolved Fluorescence

Fluorescence time traces were measured using time-correlated single-photon counting (TCSPC) using a HydraHarp 400 (PicoQuant) module attached to a MicroTime 200 confocal microscope (PicoQuant GmbH) with a 50×/0.45 NA objective (SLMPlan). An NKT SuperK Extreme pulsed (2.9–4 MHz) white light continuum laser with the SuperK Select acousto-optical tunable filter (AOTF) was used for excitation at 480 nm. A 482/12 bandpass filter was used to remove parasitic frequencies from the source. A Z488 RDC dichroic was used to reflect the excitation light into the objective, and a 488 nm notch filter was used to prevent the excitation light from reaching the detector. A detector pinhole of 150 μm was used. The detector used was a Single-Photon Avalanche Diode (MPD PDM Series, PicoQuant). The IRF was determined by measuring the signal from a solution of Erythrosin B in water saturated with potassium iodide [25]. The samples were prepared by placing a droplet of the neat liquid or solution on a 22 × 22 mm glass coverslip.

Fluorescence decay times were also obtained using time-gated detection during the transient absorption experiments described below.

### 2.5. Nanosecond Time-Resolved Spectroscopy

Nanosecond transient absorption is measured using an in-house assembled setup. For experiments in solution, the samples were contained in 1×1 cm suprasil quartz cuvettes (Hellma). The excitation wavelength of 515 nm is generated using a tunable Nd:YAG-laser system (NT342B, Ekspla) comprising the pump laser (NL300) with harmonics generators (SHG, THG) producing 355 nm to pump an optical parametric oscillator (OPO) with SHG connected in a single device. The laser system is operated at 5 Hz repetition rate. Probe light, running at 10 Hz, is generated using a high stability short arc xenon flash lamp (FX-1160, Excelitas Technologies) with a modified PS302 controller (EG&G). The probe light is split into a signal and a reference beam with a 50/50 beam splitter and focused on the entrance slit of a spectrograph (SpectraPro-150, Princeton Instruments). The probe beam (A = 1 mm${}^{2}$) is passed through the sample cell orthogonally overlapped with the excitation beam, which illuminates a 1 mm × 1 cm area of the front of the cell. The excitation power was measured at the back of the sample holder with no sample and was attenuated using a polarizing beamsplitter. The reference beam is used to normalize the signal for fluctuations in the flash lamp spectral intensity. Signal and reference beams are recorded simultaneously with a gated intensified CCD camera (PI-MAX3, Princeton Instruments) with the adjustable gate width set to 5 ns. The timing of the excitation pulse, the flash lamp, and the gate of the camera is achieved with a delay generator (DG535, Stanford Research Systems, Sunnyvale, CA, USA). The setup is controlled with an in-house written program (LabView). By analyzing the detected spectra with laser pulse-only, no probe light, we obtained the fluorescence decay times. Global and target analysis of the transient absorption data were performed using GLOTARAN [26].

### 2.6. Picosecond Time-Resolved Absorption

Thin films were prepared by dispensing a drop of pure compound in the center of a 22 × 22 mm coverslip with a thickness of 170 μm, placing a second similar coverslip on top, and then applying a small amount of pressure to distribute the liquid between the coverslips. An absorbance of approximately 1.2 was achieved using this method. Samples in solution were measured through 2 mm quartz cuvettes, and their absorbance was of approximately 1.

Time-resolved visible spectroscopy was performed using a Ti:sapphire laser (Spectra-Physics Solstice Ace, 800 nm, 2 mJ/pulse, 100 fs fwhm, 3 kHz). Pump pulses at 525 nm (energies 1 μJ for samples in solution or 0.1 μJ for thin film samples) were generated using an optical parametric amplifier (OPA-C, Spectra Physics). In short, 50% (1 mJ/pulse) of the amplified 800 nm output is split into signal and idler using difference generation through a BBO crystal, and then sum-frequency mixing of the signal with the 800 nm beam using a type II BBO crystal provides the pump pulses. A chopper in the pump path was used to block half of the pump pulses.

Five percent of the 800 nm beam (100 μJ) is passed through an computer-controlled adjustable delay stage, attenuated using a neutral density filter, and focused a small distance behind a 1 mm sapphire plate to generate the white light probe beam via supercontinuum generation. Both the 525 nm pump and white light probe pulses are overlapped at the sample, and the transmitted white light is imaged onto the entrance slit of a Shamrock spectrograph (Andor) and dispersed onto a 512 pixel CCD camera. The difference absorption is calculated as $\Delta A=-log_{10}\left(I_{p}/I_{0}\right)$, where $I_{p}$ is the intensity of the transmitted probe when both the pump and the probe pass through the sample, and $I_{0}$ is the transmitted intensity of the probe when the pump is blocked by the chopper.

A half-wave plate was used to set the relative polarization between the pump and the probe. The anisotropy was calculated using the result of two measurements: one in which the pump and the probe pulses have parallel polarizations ($\Delta A_{\|}$) and a measurement in which their polarizations are perpendicular to each other ($\Delta A_{⊥}$). The anisotropy (*r*) is calculated according to Equation (1).

(1) $$r=\frac{\Delta A_{\|}-\Delta A_{⊥}}{\Delta A_{\|}+2\Delta A_{⊥}}$$

The global and target analyses of the transient absorption data were performed using GLOTARAN [26].

### 2.7. Computation

For the quantum chemical calculations, 3,6-dimethoxy s-tetrazine ($C_{1}C_{1}$) and 3-chloro-6-methoxy-s-tetrazine ($ClC_{1}$) were used as model compounds of the dialkoxy and chloroalkoxy series, respectively. Ground and excited state geometry optimizations of $ClC_{1}$ and $C_{1}C_{1}$ were performed with the ωB97XD hybrid density functional and the cc-pVTZ basis set using the Gaussian 16 program [27]. Frequency calculations were performed at the optimized geometries, and the absence of negative frequencies confirms that the structures represent local minima. The CPK molecular volumes of these structures were calculated using Spartan18 (Wavefunction Inc., Irvine, CA, USA).

Multi-reference methods were implemented using OpenMolcas [28]. Single-point MS-CASPT2(14,10) calculations were performed a the ωB97XD/cc-pVTZ energy minima. The ANO-RCC-VTZP basis was used. The active spaces include 4 *n*, 3 π, and 3 $\pi^{*}$ orbitals (Figures S15 and S16). For minimum-energy crossing point (MECP) optimizations, the same active space was used, and the basis set was reduced to ANO-RCC-VDZP during the optimization in order to be computationally efficient. The electric transition dipole moments, oscillator strengths, and spin–orbit coupling between different excited states were calculated by applying the RAS state interaction method to the MS-CASPT2 wave functions calculated at the different minima with the ANO-RCC-VTZP basis and averaging over the first 8 roots.

## 3. Results

### 3.1. Rheology

Rheology measurements of four fluorescent liquid derivatives were performed by steady shear mode at T=20°C (see Table 1). The measured viscosities show a clear correlation with the length of two alkyl side chains attached to the tetrazine core. The compound with the shortest side chains $C_{8}C_{8}$ turns out to be the least viscous of the studied derivatives with 33.8±2.3mPa·s. Note this value is lower than the reported value in a previous study (η=58mPa·s) [11]. For the longest side chains $C_{16}C_{16}$, we obtained the highest viscosity, with 85.3±2.4mPa·s. Thus, an increase in the length of alkyl side chains led to an increase in the viscosity, possibly due to an increase in the probability of entanglement. When the symmetry of the arms is broken, i.e., for $C_{8}C_{16}$ or ${\mathrm{ClC}}_{16}$, the viscosities were found to stand in between symmetric derivatives and not very different from each other, 46.5±2.3mPa·s and 47.9±2.3mPa·s, respectively. The effect of chain length on viscosity was found to be more pronounced when the molecule had symmetry in its arms. A previous study reports a shear thickening effect for low shear rate for the $C_{8}C_{8}$ derivative [11]. In the present work, we addressed only the Newtonian regime and no information is obtained about a possible shear-thickening effect of fluorescent liquid tetrazines for lower shear rates (Indeed, the measured torque is too low (<40 nN·m) for lower shear rates ($\dot{\gamma }<5\;s^{-1}$).).

### 3.2. Absorption and Fluorescence Spectroscopy

**Figure 1:**
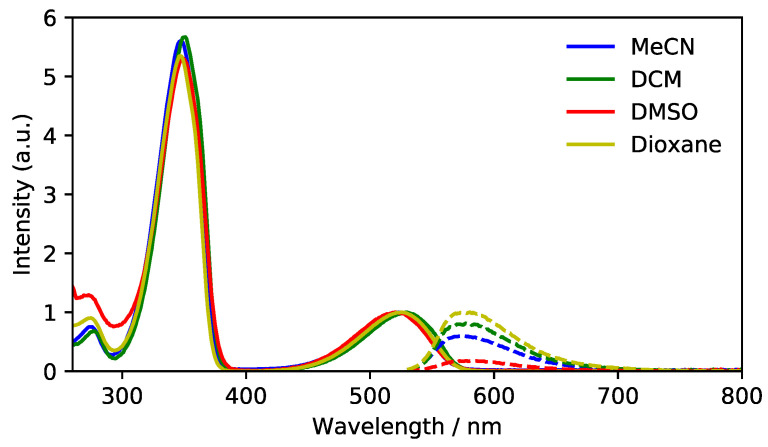
Absorption and emission spectra of $C_{16}C_{16}$ in four different solvents. The absorption spectra were normalized relative to the ${n\pi }^{*}$ absorption. The emission intensities were normalized relative to the maximum emission in dioxane, corrected for the differences in absorption of the samples, and multiplied by the solvent refractive index squared so that the maximum intensities reflect relative $\phi_{f}$.

As a representative example, absorption and fluorescence spectra of $C_{16}C_{16}$ in solvents of different polarity are shown in Figure 1. The rest of the absorption and emission spectra can be found in the SI (Figure S7). For the series of compounds, fluorescence quantum yields and fluorescence lifetimes are given in Table 2. The lifetimes measured using time-gated detection (in the same experiment as nanosecond transient absorption) and time-correlated single photon counting are in close agreement. Fluorescence quantum yields were measured for the compounds in solution. Quantum yields in the neat liquids were estimated as $\phi_{f}=k_{r}\tau$ using the radiative rate constants calculated from the lifetime and $\phi_{f}$ in DCM.

### 3.3. Nanosecond Transient Absorption

**Figure 2:**
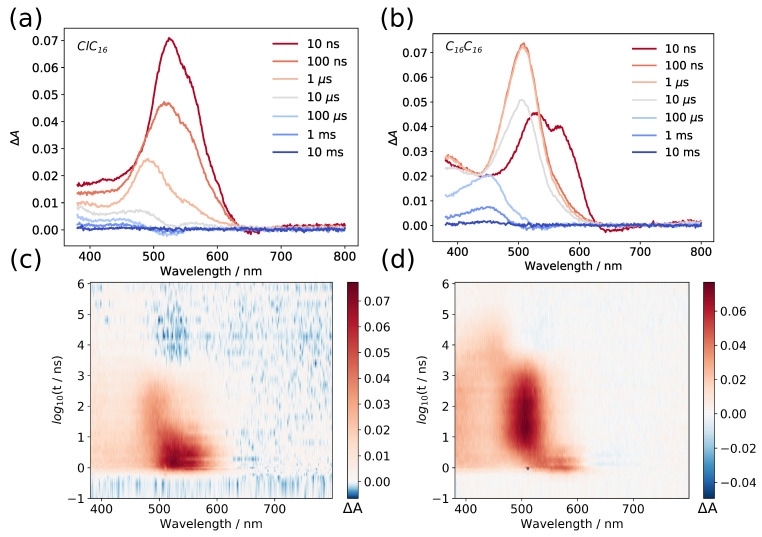
Nanosecond time-resolved spectra of (**a**) $ClC_{16}$ and (**b**) $C_{16}C_{16}$ at a few selected delays, and their respective transient matrices (**c**,**d**).

Global fitting the transient absorption matrix using three components is enough to obtain an adequate fit (Table 3, Figure 2). The spectrum associated with each of the components can be found in the SI (Figures S8–S11), and the absorption maxima of each component is given in Table 4.

The faster decay is consistent with the fluorescence lifetime, so this component is assigned to the $S_{1}$ state. For the dialkoxy series, this lifetime is of 30 ns, and it is of 124 ns for $ClC_{16}$. The longer fluorescence lifetime of $ClC_{16}$ is due to its slower rate of intersystem crossing in the chloroalkoxy compounds, which explains their higher fluorescence quantum yield.

The lifetime of the second component is dramatically reduced when oxygen is allowed to enter the solution (Figure S12, Table S1), so we assign this component to the $T_{1}$ state. Moreover, the spectrum is similar to that recently assigned to the triplet state in amino-substituted tetrazines [29]. This lifetime is affected by the excitation power Figure S12, Table S1), indicating that this species decays via a triplet-triplet quenching pathway.

The spectrum of the third component, which appears to be formed in small quantities, has an absorption consistent with the absorption spectrum of the tetrazine radical anion, which has been previously experimentally measured for $ClC_{1}$ [30]. The formation of the tetrazine cation from two triplet molecules would be highly energetically unfavorable ($E_{Cation}+E_{Anion}-2E_{T1}$ = 4 eV for $C_{1}C_{1}$ at the ωB97XD/cc-pVTZ level of theory), so the source of the electron remains unclear.

### 3.4. Picosecond Transient Absorption

Ultrafast transient absorption spectra in a time window between 0.1 ps and 3 ns show a single absorption band in the 500 nm to 600 nm region, which is ascribed to the $S_{1}$ state.

**Figure 3:**
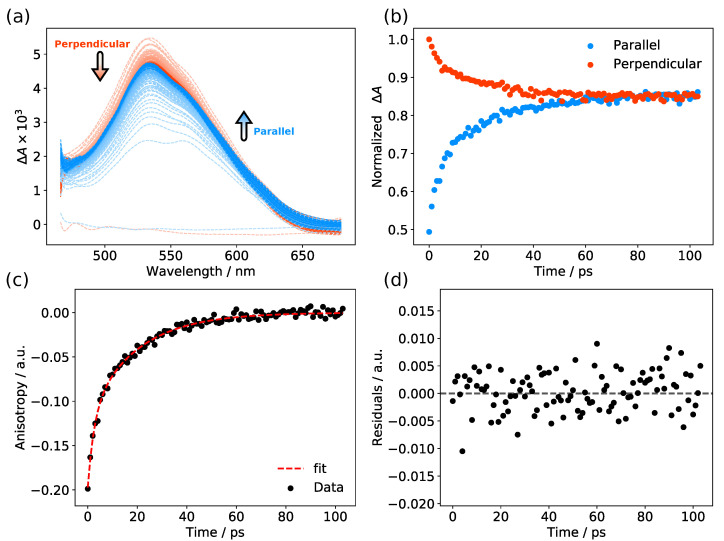
Picosecond time-resolved absorption and anisotropy of $ClC_{16}$ in DCM. (**a**) ps time-resolved absorption of $ClC_{16}$ in DCM measured with parallel and perpendicular relative polarization of the pump and probe. (**b**) The difference absorption integrated over the absorption band (ΣΔA) vs delay time a parallel and perpendicular pump/probe configuration. (**c**) The decay of the anisotropy fitted to a single exponential decay. (**d**) Residuals of the fit.

By monitoring the anisotropy decay of the transient absorption (Figure 3), we obtain insight into the rotational dynamics of the molecule in solution. In the di-alkoxy neat liquid we observe a decay of the anisotropy faster than what one would expect for the rotational decorrelation in the viscous environment that the molecules will experience as a neat liquid (Page 9, Table 1 and Table 5).

To obtain a consistent estimate of the molecular volumes of the compounds, we used the CPK volumes calculated using Spartan18 for the optimized geometries. For $C_{8}C_{8}$, this volume is calculated to be 383 Å${}^{3}$, which agrees well with the molecular volume from the density of the neat liquid (377 Å${}^{3}$) [11]. The volumes were used to calculate the rotational diffusion time constants predicted by the Stokes–Einstein equation (Equation (2)) for a sphere of the same volume [31].

(2) $$\tau_{rot}^{sphere}=\frac{\eta V}{k_{B}T}$$

The rotational decorrelation time constants for a perfectly spherical molecule in a solvent with the viscosity of DCM calculated using the Einstein–Stokes equation are 1.8 to 3 times as large as the measured values (Table 5). As the measured molecules are not spherical, a deviation from the Einstein–Stokes value is expected. The order of magnitude of these rates is, however, what we would expect to see if the main anisotropy decay pathway is the randomization of the orientations via rotational diffusion.

The decay of the anisotropy in the neat films occurs with a similar order of magnitude as the decay in DCM (Table 5). This is surprising because in the neat liquid the molecules will experience an environment with the viscosity of the neat liquid (Table 1), and rotational decorrelation is expected to be much slower, as calculated using the Einstein–Stokes equation (Table 5). An explanation is proposed in the Discussion (Section 4).

### 3.5. Computation

In order to explore the electronic structural differences between the dialkoxy and chloro/alkoxy compounds, we investigated the electronic structure of 3,6-dimethoxy s-tetrazine ($C_{1}C_{1}$) and 3-chloro-6-methoxy s-tetrazine ($ClC_{1}$) in vacuum. We begin with the results of geometry optimizations and TD-DFT calculations at the ωB97XD/cc-pVTZ level of theory. The vertical transition energies at the different minima can be found in the SI (Tables S2 and S5, and the geometric parameters are tabulated in Tables S8 and S9).

The coordinate system is defined such that the s-tetrazine core lies in the yz plane, with the CO bond lying along the z-axis. Both model compounds are predicted to have an n-type HOMO and a $\pi^{*}$ type LUMO. The first transition corresponds to the $n\pi^{*}$ state, which is calculated to be at 520 nm for $ClC_{1}$ and 530 nm for $C_{1}C_{1}$ in agreement with experiment. The $S_{1}\leftarrow S_{0}$ transition is only weakly allowed for both compounds because of a small electronic transition dipole moment (ETDM) along the x-axis (f = 0.005).

The strong absorption band near 340 nm corresponds to the (HOMO−1)→LUMO$\pi \pi^{*}$ band. This is calculated to be the $S_{2}\leftarrow S_{0}$ transition of $C_{1}C_{1}$ (calc: 303 nm, exp: 350) and the $S_{3}\leftarrow S_{0}$ transition for $ClC_{1}$ (calc: 284 nm, exp: 330). The ETDM of this transition lies in the ZY plane and has a moderate oscillator strength (f = 0.056).

The ground-state TDDFT vertical excitation calculations are in good agreement with the steady state absorption for the $n\pi^{*}$ transition, but overestimate the vertical energy of the $\pi \pi^{*}$ state.

The vertical $S_{1}S_{0}$ transition from the first excited state at the $S_{1}$ minimum is calculated to be 604 nm (exp: 590) for $C_{1}C_{1}$ and 595 nm (exp: 566) for $ClC_{1}$, which corresponds to the measured emission.

The time-resolved absorption measured in our pump-probe experiments can be correlated to the vertical transition energies at the sufficiently long-lived intermediates explored by the molecule during its excited state dynamics. In addition, the time-resolved anisotropy measurements allow us to determine the relative angle between the electronic transition dipole moments of the pumped $S_{1}\leftarrow S_{0}$ transition and the probed transitions.

At the TD-DFT level of theory, the $S_{2}\leftarrow S_{1}$ transition at the $S_{1}$ minimum is calculated to occur at 661 nm for $ClC_{1}$ and 543 nm for $C_{1}C_{1}$. We do not observe such a large difference in the absorption maxima of the two groups of compounds in our transient absorption spectra. The nature of this transition is $n\pi^{*}\to \pi \pi^{*}$, which at the MS-CASPT2 level (see later discussion) is shown to have a low oscillator strength and an ETDM parallel to the ETDM for $S_{1}\leftarrow S_{0}$. The transition $S_{3}\leftarrow S_{1}$ is predicted at 488 nm for $ClC_{1}$ and at 520 nm for $C_{1}C_{1}$.

The $T_{1}$ state is of ${}^{3}n\pi^{*}$ character at the $S_{0}$, $S_{1}$, and $T_{1}$ minima. At the $T_{1}$ minimum, the energy difference between the $S_{0}$ and $T_{1}$ states is 1.47 eV (TDDFT)/1.37 (CASPT2) for $C_{1}C_{1}$, a bit smaller than the reported phosphorescence energy [17]. The vertical transition $T_{2}\leftarrow T_{1}$ is calculated to occur at 716 nm for $ClC_{1}$ and at 930 nm for $C_{1}C_{1}$. The $T_{2}$ state has ${}^{3}\pi \pi^{*}$ character, and the $T_{2}\leftarrow T_{1}$ transition is forbidden for both compounds (f = 0.000). The $T_{3}\leftarrow T_{1}$ transition is predicted at 530 nm (f = 0.051) for $C_{1}C_{1}$ and 558 nm (f = 0.029) for $ClC_{1}$. The strong excited state absorption that we see in the ns time-resolved experiments is likely due to this transition, observed around 500 nm.

The results at the MS-CASPT2 level paint a similar overall picture (Tables S3 and S6), but the $T_{2}$ state is moved up in energy by about 1 eV. This higher energy breaks the degeneracy at the $S_{1}/T_{2}$ crossing optimized at the CASSCF level. The CASPT2 wave functions can also be used to compute the oscillator states and ETDM between the different excited states by using the RAS State Interaction method (RASSI) (Tables S4 and S7). According to these results, the lowest-energy transition that should result in an anisotropy value of −0.2 are the $S_{5}\leftarrow S_{1}$ for $C_{1}C_{1}$ (at 479 nm) and the $S_{4}\leftarrow S_{1}$ for $C_{1}C_{1}$ (464 nm). In both cases, the state corresponds to a π state, where the $\pi^{*}$ LUMO orbital becomes doubly occupied, and the transition dipole moment is along the z-axis.

To explain why the rate of ISC is greater for the dialkoxy than for the chloro/alkoxy compounds, we first calculated the spin–orbit coupling matrix elements (SOC). The SOC between $S_{1}$ and $T_{1}$ is calculated to be 0.2 cm${}^{-1}$ at the $S_{1}$ minimum for both compounds. On the other hand, we observe a moderate SOC (9.7 cm${}^{-1}$ for $C_{1}C_{1}$, 7.2 cm${}^{-1}$ for $ClC_{1}$) between the ${}^{1}n\pi^{*}$ and the ${}^{3}\pi \pi^{*}$$T_{2}$ state. This is consistent with the El Sayed rules, which predict a strong SOC when the transition involves an electron moving from a π to an *n* orbital, as is the case for the ${}^{1}n\pi^{*}\to^{3}\pi \pi^{*}$.

**Figure 4:**
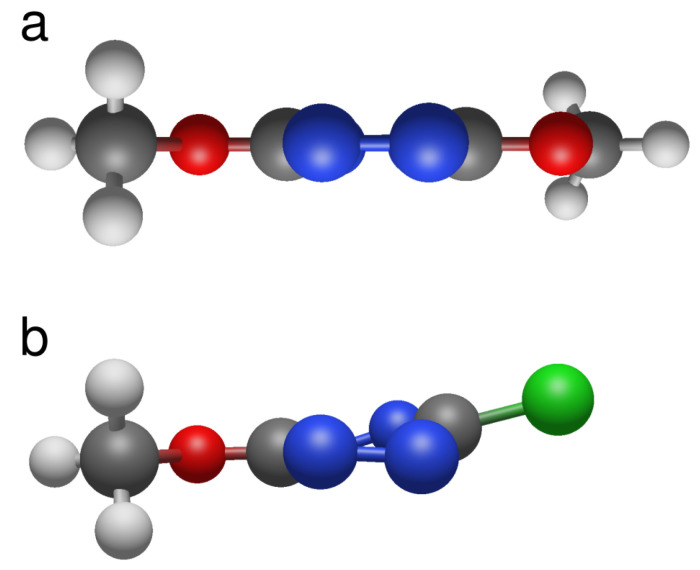
$S_{1}/T_{2}$ minimum energy crossing points of $C_{1}C_{1}$ (**a**) and of $ClC_{1}$ (**b**).

Based on the hypothesis that ISC occurs efficiently at geometries near the $S_{1}$/$T_{2}$ minimum-energy crossing point (MECP), we located these at the CASSCF(14,10)/ANO-RCC-VDZP level of theory. The energy needed to reach the $S_{1}/T_{2}$ crossing is 8.5 kcal/mol for $C_{1}C_{1}$ and 9.7 kcal/mol for $ClC_{1}$ (no solvation model). In addition to the higher energy required for the crossing, we can see that reaching the $ClC_{1}$ MECP requires a expanding and twisting the tetrazine core, while the core of $C_{1}C_{1}$ only has to expand (Figure 4; Tables S8 and S9). At the $S_{1}/T_{2}$ crossing points there is little difference between the SOC’s for $ClC_{1}$ and $C_{1}C_{1}$ (Table 6). Therefore, we attribute the more efficient ISC in the dialkoxy compounds compared to the chloro/alkoxy compounds to the relatively higher energy of the crossing point in the latter.

## 4. Discussion

Fluorescence in a neat compound, either as a solid or as a liquid, is often spectrally different from that of the compound diluted in solution, and it is commonly much weaker due to the occurrence of quenching processes that are related to the presence of low-energy dark states that result from electronic coupling between chromophores. In the series of compounds studied in the present work however, the fluorescence parameters in the neat liquids do not differ much from those in solution (Table 2). The spectra are only slightly shifted, and the quantum yields (which are controlled by the nonradiative decay rates) are similar. For the asymmetric $ClC_{8}$, it was found previously that the quantum yield in the neat liquid was lower than in DCM solution. For the analogous compound with the $C_{16}$ chain, we find that this quenching effect is smaller, possibly because the average intermolecular distance is increased. For $ClC_{16}$ and $C_{16}C_{16}$, we compare the quantum yields in three solvents. In both cases, the fluorescence quantum yield decreases with increasing solvent polarity, going from dioxane to DCM to acetonitrile. Interestingly, for $C_{8}C_{16}$ and for $C_{16}C_{16}$ the quantum yields in the neat liquids are a bit higher than in DCM, which is likely associated with the medium polarity effect.

The data in solution clearly reveal the systematic trend that nonradiative decay in the dialkoxy compounds is faster than in the chloro/alkoxy systems. The transient absorption results show that the major process that competes with fluorescence is intersystem crossing [17]. The calculations indicate that this involves a crossing from $S_{1}$ to $T_{2}$. The situation in which crossing from an $S_{1}$ to a $T_{1}$ state of the same character by using a $T_{2}$ state with a different symmetry as an intermediate has been previously observed in other molecules [32]. Note that the occurrence of efficient fluorescence of substituted tetrazines is related to the nature of the frontier orbitals [10]. A relationship was found between the energy difference between the HOMO-1 and HOMO, and the fluorescence quantum yield. Here, we suggest that the underlying reason for this observation is the relationship between the first singlet excited state (${}^{1}n\pi^{*}$) and the ${}^{3}\pi \pi^{*}$ and ${}^{3}n\pi^{*}$ states. In accordance with El-Sayed’s rules, the relative ordering and large spin-orbit coupling between the $S_{1}$${}^{1}n\pi^{*}$ and the $T_{2}$${}^{3}\pi \pi^{*}$ leads to a rapid non-radiative decay into the triplet [33,34].

For the dialkoxy and chloroalkoxy compounds, the $T_{1}$ state is ${}^{3}n\pi^{*}$, and the $T_{2}$${}^{3}\pi \pi^{*}$ is higher in energy. However, small distortions of the tetrazine core are enough for an $S_{1}/T_{2}$ crossing to occur (Tables S8 and S9). According to our calculations the energy barrier required to reach the $S_{1}/T_{2}$ crossing is 8.5 kcal/mol for $C_{1}C_{1}$ and 9.7 kcal/mol for $ClC_{1}$ when no solvation model is used. If we apply the Erying equation at 300 K and setting the transmission coefficient κ to 1, the expected barriers to reproduce the observed non-radiative decay rates are 7.3 kcal/mol (28 μs${}^{-1}$) and 8.5 kcal/mol (4.2 μs${}^{-1}$), respectively. This shows that the energy barrier is systematically overestimated by 1.2 kcal/mol, but there is an excellent agreement in the difference of the barrier between the two compounds.

Applying an SCRF solvation model using acetonitrile as the solvent stabilizes the ${}^{3}\pi \pi^{*}$ relative to the other states, and decreases the barrier between the $S_{1}$ minimum and the $S_{1}/T_{2}$ crossing point by 0.3 kcal/mol for $C_{1}C_{1}$. This lowering of the energy required for reaching the crossing and explains the solvent effect on the fluorescence quantum yields. Thus, formation of the triplet occurs via an activated process in which the molecules distorts towards this crossing point. In the context of the present work, it is important to note that the geometry changes required are rather small, and not hindered by the viscosity of the medium.

The time-resolved anisotropy measurements in solution show a clear correlation between the anisotropy decay rates and the size of the molecules. Thus, it is reasonable to attribute the decay to tumbling of the molecules in the solution. In the neat liquid, however, the anisotropy decay rates are similar to those in dichloromethane, while the viscosities are much higher (Table 1). Apparently, other mechanisms contribute to anisotropy decay in the neat liquids. One plausible explanation is that the fast decay of the anisotropy in the neat film is due to energy transfer between neighboring molecules. This fast exciton diffusion leads to a rapid depolarization of the excited state population, and manifests itself as a fast decay of the anisotropy.

A model derived by Huber [35,36] predicts the anisotropy decay r(t) as described in Equations (3) and (4), in which *c* is the concentration (molecules nm${}^{-3}$), $\tau_{0}$ the fluorescence lifetime, and $R_{0}$ is the Förster radius (nm), defined in Equation (5).

(3) $$r\left(t\right)=r_{0}e^{-2\gamma^{\prime }{\left(\frac{t}{\tau_{0}}\right)}^{1/2}}$$

(4) $$\gamma^{\prime }=\frac{2\pi^{3/2}}{3\sqrt{2}}cR_{0}^{3}$$

(5) $$R_{0}=0.02108{\left[\frac{\kappa^{2}\phi_{f}J^{\lambda }}{n^{4}}\right]}^{\frac{1}{6}}$$

The spectral overlap integral $J^{\lambda }$ is given by Equation (6).

(6) $$J^{\lambda }=\int \bar{I}\left(\lambda \right)\epsilon \left(\lambda \right)\lambda^{4}\;d\lambda ;\int \bar{I}\left(\lambda \right)d\lambda =1$$

$\bar{I}\left(\lambda \right)$ is the normalized emission spectrum and ϵ(λ) is the molar absorption coefficient. κ is the orientation factor, which is 2/3 when the neighbouring acceptor molecules are randomly oriented [37].

The spectral overlap integral calculated using the absorption and emission data of $C_{8}C_{8}$ in DCM is $J^{\lambda }$ = 5.2 × 10${}^{12}$ M${}^{-1}$ cm${}^{-1}$ nm${}^{4}$. Plugging in the refractive index n=1.424 and the measured quantum yield $\phi_{f}$, an $R_{0}$ of 1.31 nm is obtained for $C_{8}C_{8}$ in DCM. For $C_{8}C_{8}$ the density is known, so we can estimate the average distance between two molecules as ~1 nm, similar to $R_{0}$, leading to a predicted Förster energy transfer rate of 2.2 × 10${}^{8}$ s${}^{-1}$.

**Figure 5:**
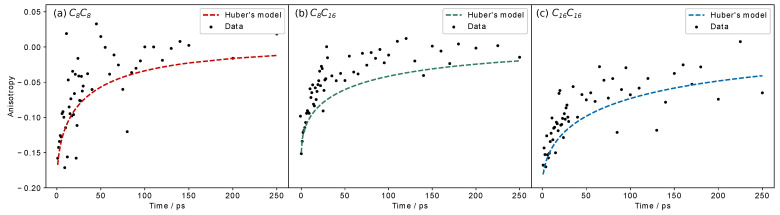
The decay of the anisotropy in the dichloroalkoxy neat films, plotted against the curves predicted by the Huber model.

Inserting these computed values, 30 ns as the fluorescence lifetime, and setting the density to $\rho =\frac{1}{V_{CPK}}$, where $V_{CPK}$ are the calculated volumes (5), we obtain curves that fit reasonably well with our observed anisotropy decay. The model and the measured decay are plotted in Figure 5.

## 5. Conclusions

We have studied a set of bis-alkoxy and chloro/alkoxy tetrazines which are viscous liquids at ambient temperature owing to the branched alkoxy chains. The viscosity of the liquids increases with the size of the substituents from 28 mPa · s for ClC8 [11] to 85 mPa · s for $C_{16}C_{16}$. All compounds studied are fluorescent in solution and in the neat liquid state. The stronger fluorescence of the chloro/alkoxy systems in solution compared to the bis-alkoxy systems is caused by slower inter system crossing. The intersystem crossing occurs from $S_{1}$ (${n\pi }^{*}$) to $T_{2}$ (${\pi \pi }^{*}$) and the thermal activation energy to reach the crossing point is smaller in the chloro/alkoxy tetrazines. Moreover, the activation energy is weakly dependent on the solvent polarity, giving rise to stronger fluorescence in less polar solvents.

## Figures and Tables

**Table 1:** Viscosities of four fluorescent liquid derivatives.

Liquid	Viscosity η [mPa·s]
$C_{8}C_{8}$	33.8±2.3
${\mathrm{ClC}}_{16}$	47.9±2.3
$C_{8}C_{16}$	46.5±2.3
$C_{16}C_{16}$	85.3±2.4

**Table 2:** Fluorescence parameters of the studied compounds: quantum yield $\phi_{f}$, fluorescence decay time $\tau_{f}$, radiative decay rate constant $k_{r}$, and non-radiative decay rate constant $k_{\mathrm{nr}}$.

	Solvent	$\phi_{f}$	${\tau_{f}}^{\;a}/\mathrm{ns}$	${\tau_{f}}^{\;b}/\mathrm{ns}$	$k_{r}\;\left(\times \;10^{6}s^{-1}\right)$	$k_{\mathrm{nr}}\left(\times \;10^{6}s^{-1}\right)$
$C_{8}C_{8}$	DCM	0.069 ^*c*^	33	34	2.1	28
$C_{8}C_{8}$	neat	0.08 ^*d*^		38	2.1 ^*d*^	24 ^*d*^
$C_{8}C_{8}$	neat	0.068 ^*e*^		33 ^*e*^	2.1 ^*e*^	28^*e*^
$C_{8}C_{16}$	DCM	0.080 ^*c*^	36	35	2.2	26
$C_{8}C_{16}$	neat	0.09 ^*d*^		43	2.1 ^*d*^	$21^{d}$
$ClC_{8}$	DCM	0.33 ^*e*^	158^*e*^		2.1 ^*e*^	4.2 ^*e*^
$ClC_{8}$	neat	0.021 ^*e*^		4.6 ^*e*^	4.6 ^*e*^	213 ^*e*^
$C_{16}C_{16}$	DCM	0.068 ^*c*^	32	37	2.1	29
$C_{16}C_{16}$	MeCN	0.052 ^*c*^	31		1.7	31
$C_{16}C_{16}$	Dioxane	0.089 ^*c*^	45		2.0	20
$C_{16}C_{16}$	neat	0.11 ^*d*^		52	2.1 ^*d*^	17 ^*d*^
$ClC_{16}$	DCM	0.37 ^*c*^	151		2.5	4.2
$ClC_{16}$	MeCN	0.34 ^*c*^				
$ClC_{16}$	Dioxane	0.40 ^*c*^				
$ClC_{16}$	neat	0.10 ^*d*^		48	2.1 ^*d*^	21 ^*d*^

${}^{a}$ from time-gated fluorescence detection; ${}^{b}$ average lifetime from TCSPC (full data in Table S11 in SI); ${}^{c}$ measured in this work; ${}^{d}$ calculated assuming τ using $k_{r}=2.1$ ns; ^*e*^ from the work in [11].

**Table 3:** Lifetimes obtained from globally fitting the nanosecond transient absorption experiments using a three-step sequential model. The solvent used is DCM unless noted otherwise.

	$\tau_{1}\left(\mathrm{ns}\right)$	$\tau_{2}\left(\mu s\right)$	$\tau_{3}\left(\mathrm{ms}\right)$
$C_{8}C_{8}$	32	44	1.08
$C_{8}C_{16}$	30	40	0.95
$C_{16}C_{16}$	27	25	0.73
${\mathrm{ClC}}_{16}$	124	9	7.6

**Table 4:** Absorption maxima of the transitions measured in steady state absorption ($S_{0}$) and in nanosecond transient absorption ($S_{1}$, $T_{1}$).

	$S_{0}\lambda_{\max}\;\left(\mathrm{nm}\right)$	$S_{1}\lambda_{\max}\;\left(\mathrm{nm}\right)$	$T_{1}\lambda_{\max}\;\left(\mathrm{nm}\right)$
$C_{8}C_{8}$	529	572	507
$C_{8}C_{16}$	529	572	507
$C_{16}C_{16}$	529	572	507
$ClC_{16}$	521	527	494

**Table 5:** Time-resolved anisotropy and the calculated rotational diffusion for a sphere with the same hydrodynamic volume in DCM, using η = 0.43 mPa · s for DCM, η = 1.2 mPa · s for dioxane, and values from Table 1 for the neat liquids. T = 293 K.

	Solvent	$\tau_{r}/\mathrm{ps}$	$r_{0}$	V/ Å${}^{3}$	$\tau_{\mathrm{rot}}^{\mathrm{sphere}}/\mathrm{ns}$
$C_{8}C_{8}$	DCM	12.8±4.6	−0.23±0.02	383	0.04
$C_{8}C_{8}$	neat	25.1±2.0	−0.19±0.01		3.65
$C_{8}C_{16}$	DCM	25.0±2.6	−0.19±0.01	530	0.06
$C_{8}C_{16}$	neat	21.31	−0.13		4.57
$C_{16}C_{16}$	DCM	39.4±2.6	−0.22±0.01	677	0.07
$C_{16}C_{16}$	Dioxane	159.3	−0.211		0.20
$C_{16}C_{16}$	neat	34.5	−0.13		6.06
$ClC_{16}$	DCM	20.2±3.5	−0.20±0.01	388	0.04

**Table 6:** The largest spin–orbit coupling elements between the relevant singlets and triplets in cm${}^{-1}$ at the $S_{1}$ and $T_{1}$ TD-DFT minima, and at the minimum-energy (CAS(14,10)/ANO-RCC-VDZP) $S_{1}/T_{2}$ crossing. The spin–orbit coupling elements are calculated at the MS-CASPT2(14,10)/ANO-RCC-VTZP level, state-averaging over the lowest three roots for singlets, and the first two roots for the triplets.

	$S_{1}\Leftrightarrow T_{1}$	$S_{1}\Leftrightarrow T_{2}$	$T_{1}\Leftrightarrow S_{0}$
$C_{1}C_{1}$			
$S_{1}$	0.2	9.7	0.0
$T_{1}$	0.2	9.7	0.0
$S_{1}/T_{2}$	1.1	12.5	0.0
${\mathrm{ClC}}_{1}$			
$S_{1}$	0.2	7.2	2.9
$T_{1}$	0.2	7.2	3.3
$S_{1}/T_{2}$	0.8	12.0	4.7

## Data Availability

The experimental data used in this study are available upon request from the authors.

## Supplementary Materials

A PDF file containing the supporting images and tables can be found in the publisher’s website. Figures S1–S6: H and C NMR spectra of the studied compounds. Figure S7: Normalized absorption and emission spectra of the studied compounds in DCM. Figures S8–S11: Species-associated spectra fitted from the nanosecond time-resolved studies in DCM. Figure S12: Nanosecond time-resolved transient matrices of $C_{16}C_{16}$ in DCM. Figures S13 and S14: Energy diagrams of $C_{1}C_{1}$ and $ClC_{1}$ computed at the ωB97XD/cc-pVTZ level of theory. Figures S15 and S16: The active space of $ClC_{1}$ and of $C_{1}C_{1}$ used in the MS-CASPT2 calculations. Figure S17: Plots of the TCSPC fluorescence decays of the dialkoxy derivatives. Figure S18: Plot of he viscosity of the four fluorescent liquid derivatives as a function of shear rate. Table S1: Three-component lifetimes obtained from fitting the nanosecond time-resolved data. Table S2–S9: Energies, transitions, and and geometric parameters obtained from quantum chemical calculations. Table S10: The fluorescence lifetime of the fluorescent liquids measured in DCM using TCSPC. Table S11: The fluorescence lifetime of the neat liquids measured in DCM using TCSPC.

## Sample Availability

Samples of the compounds are available from P.A.
