# Supplementary material for: Fluorescent Liquid Tetrazines
Source: Molecules. 2021 Oct 6;26(19):6047. doi: 10.3390/molecules26196047 (PMC8512366; doi:10.3390/molecules26196047)
Supplement: Supplementary file 1 [file molecules-26-06047-s001.zip › molecules-1399021-SI.pdf]

Supplementary Materials: Fluorescent Liquid Tetrazines

Maximilian Paradiz Dominguez [1], Begüm Demirkurt [1], Marion Grzelka [2], Daniel Bonn[2], Laurent Galmiche[3], Pierre Audebert[3], and Albert M. Brouwer [1,4,*]

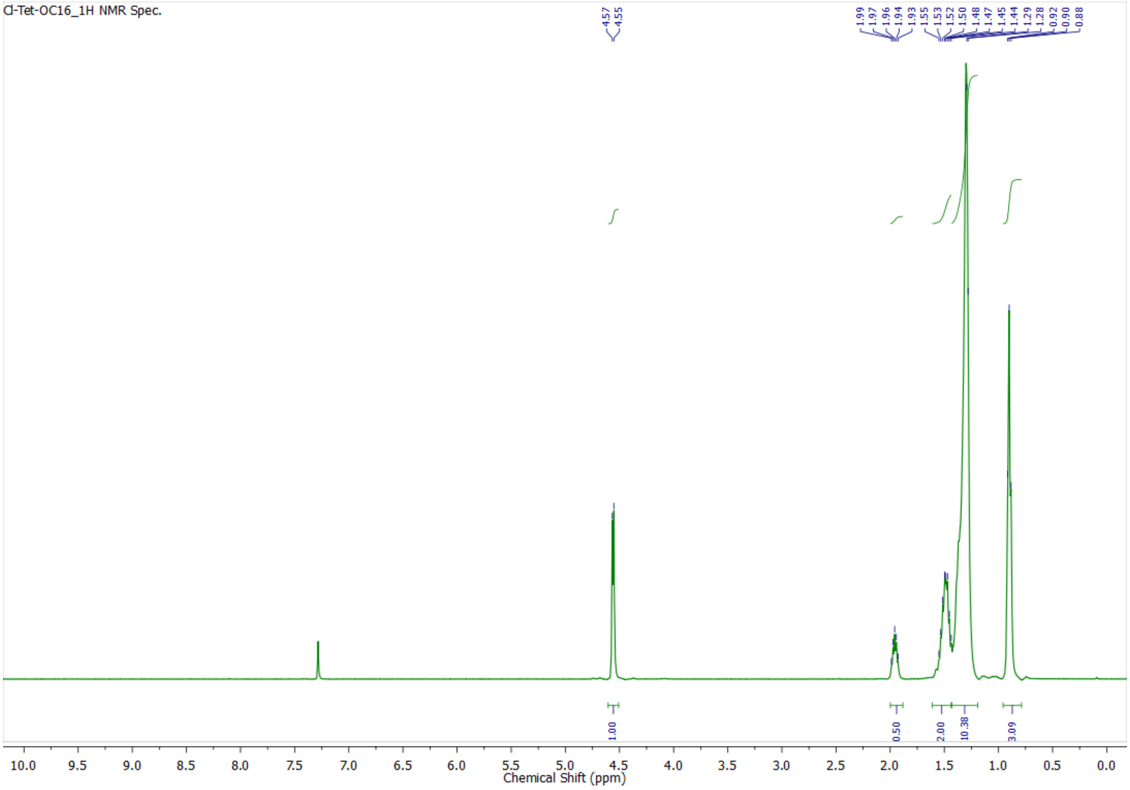

Figure S1. ^{1}H NMR spectrum of *ClC*$_{16}$

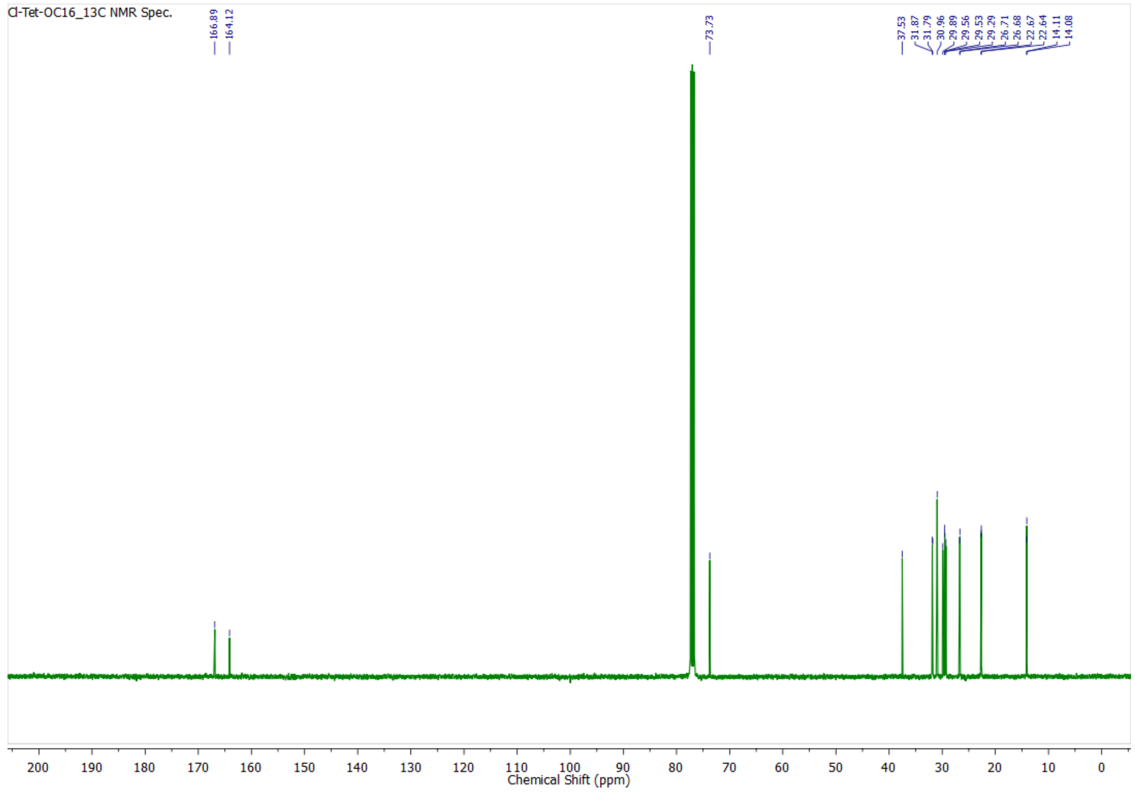

Figure S2. ^{13}C NMR spectrum of ClC_{16}

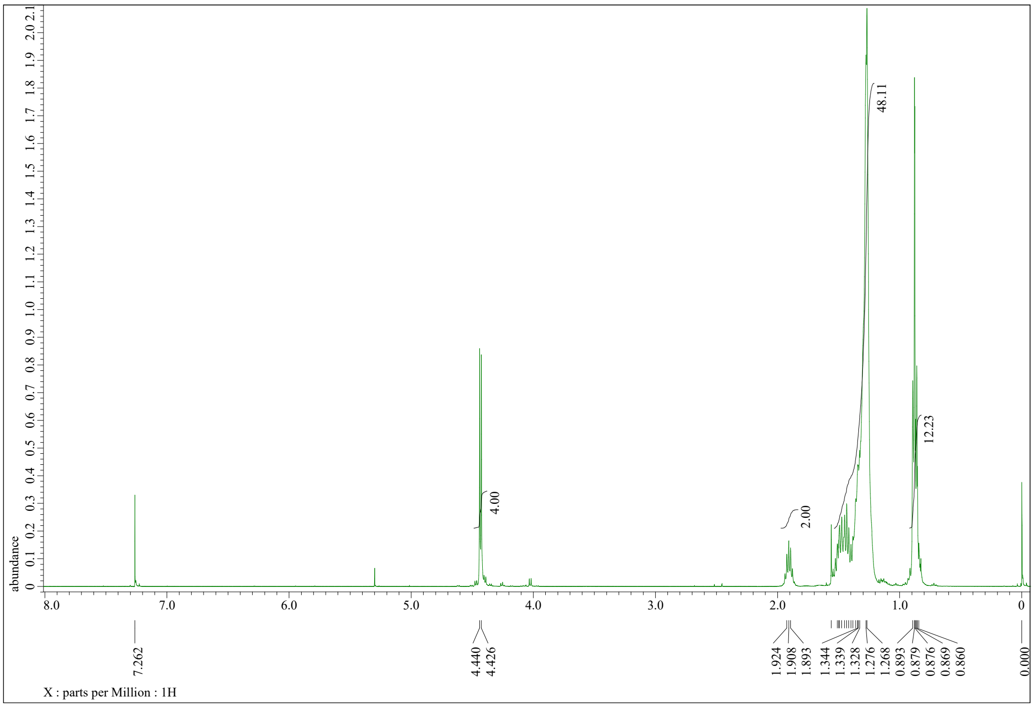

Figure S3. ^{1}H NMR spectrum of $C_{16}C_{16}$

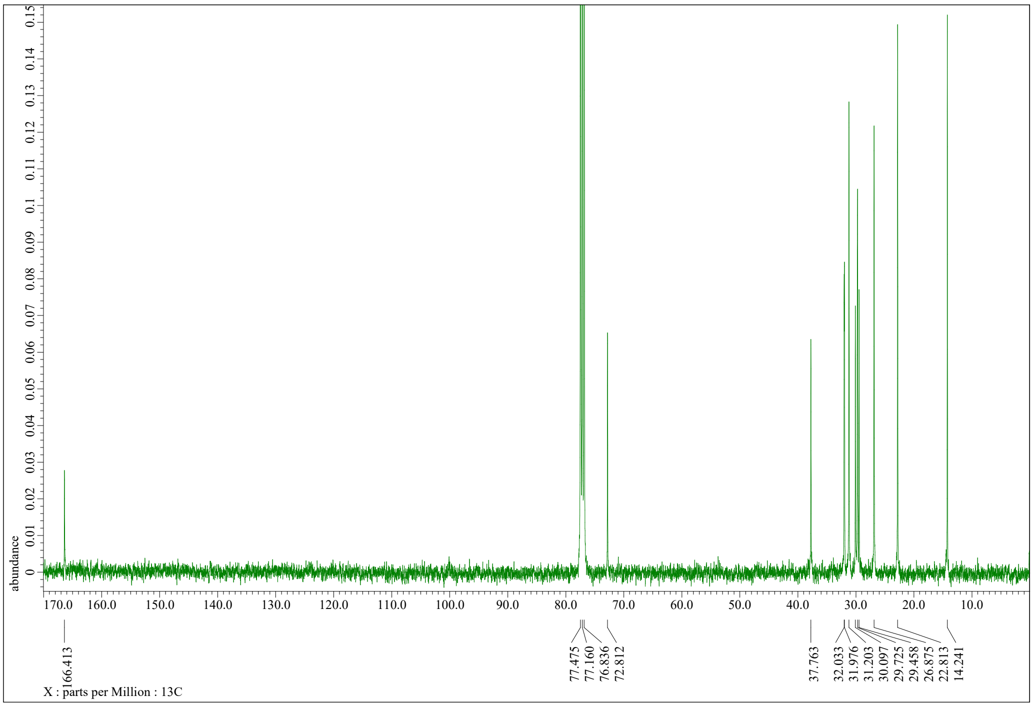

Figure S4. ^{13}C NMR spectrum of $C_{16}C_{16}$

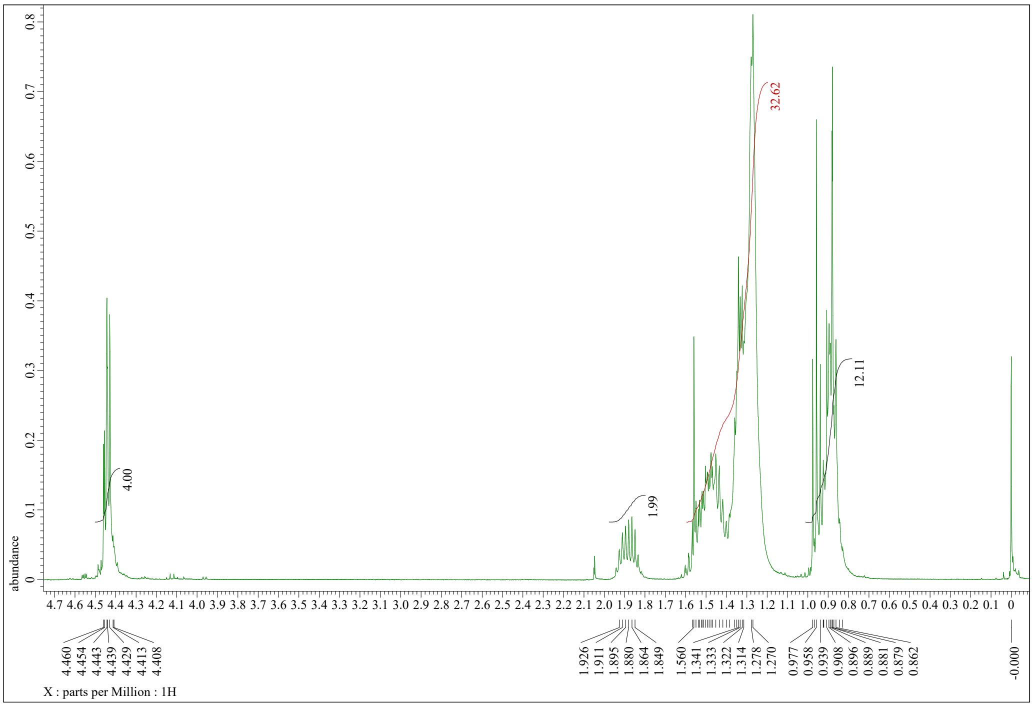

Figure S5. ^{1}H NMR spectrum of $C_{8}C_{16}$

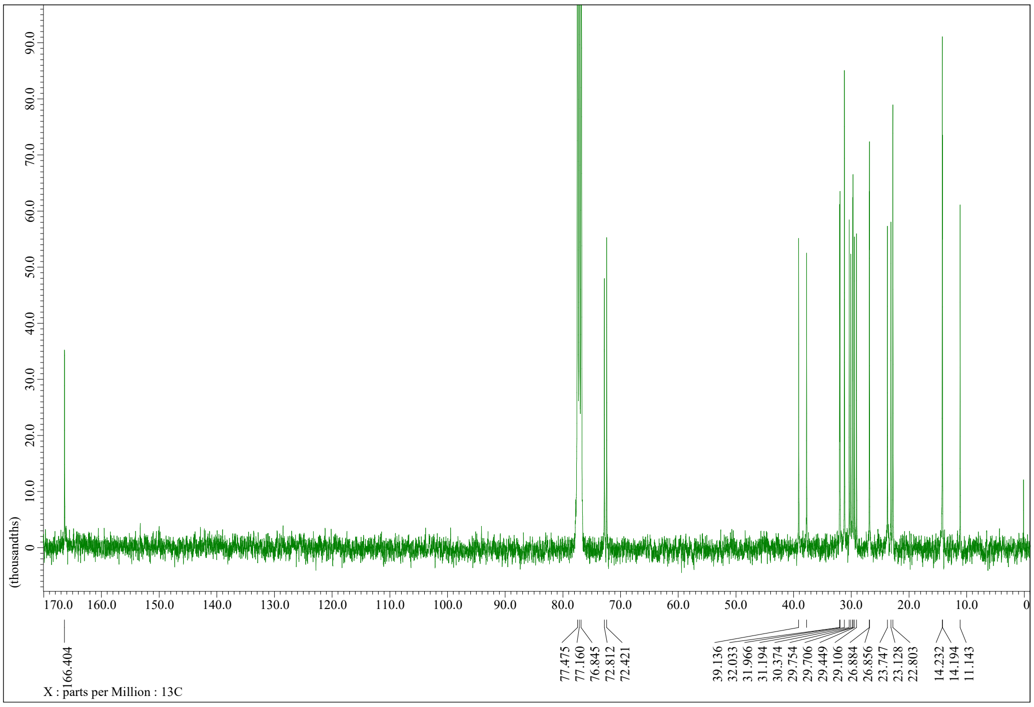

Figure S6. ^{13}C NMR spectrum of C_8C_{16}

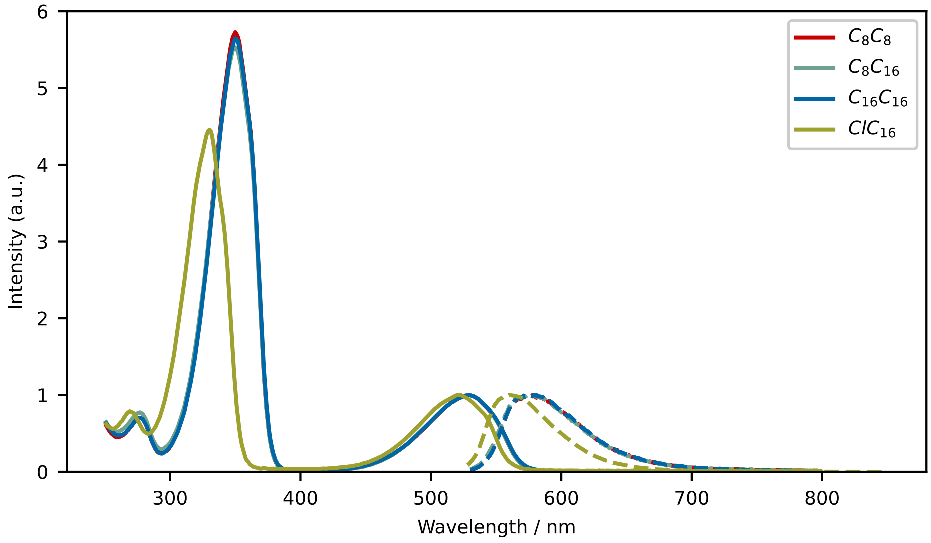

Figure S7. Normalized absorption (solid lines) and emission (dotted lines) spectra of the studied compounds in DCM.

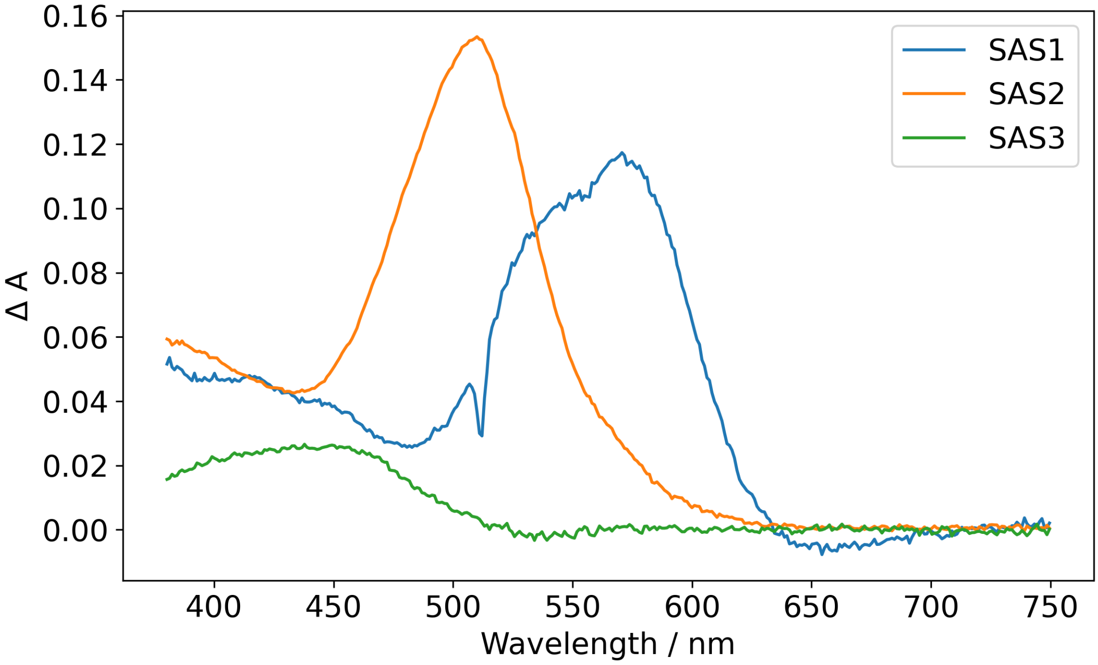

Figure S8. The species-associated spectra of C_8C_8 in DCM obtained from globally fitting the nanosecond transient absorption matrix (1 mJ pump) to a sequential model with three components.

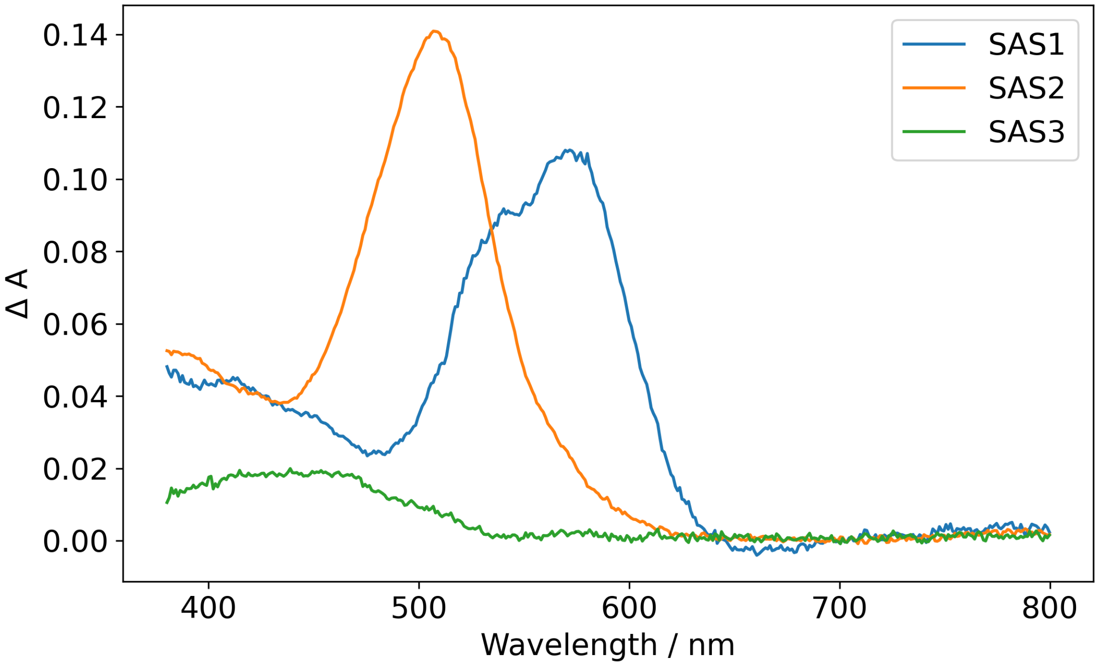

Figure S9. The species-associated spectra of C_8C_{16} in DCM obtained from globally fitting the nanosecond transient absorption matrix (1 mJ pump) to a sequential model with three components.

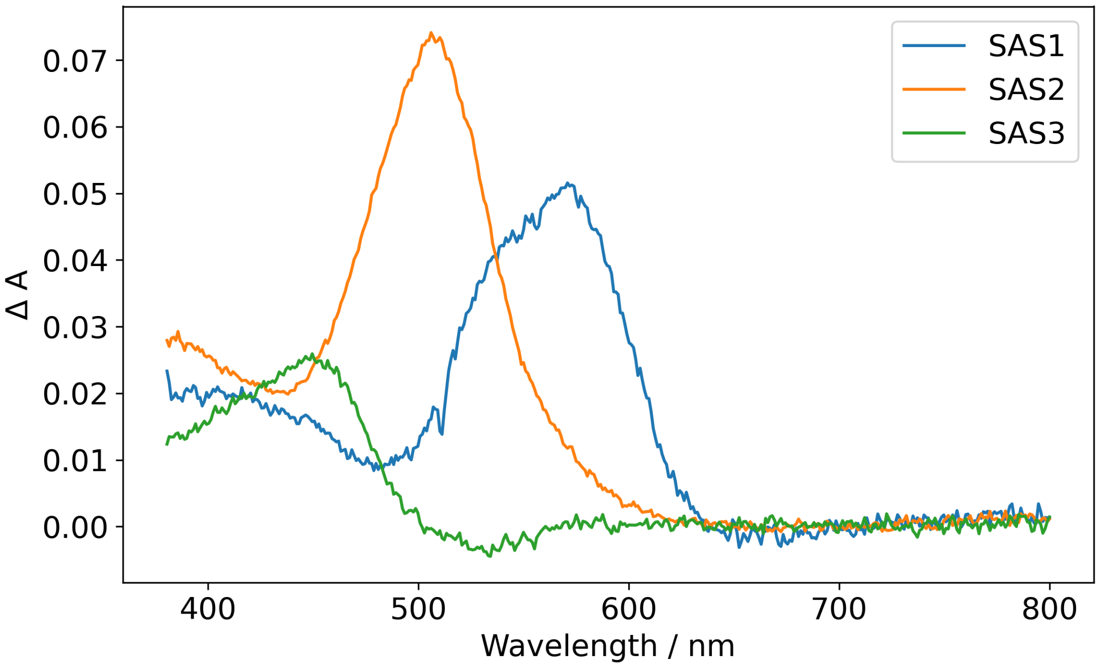

Figure S10. The species-associated spectra of $C_{16}C_{16}$ in DCM obtained from globally fitting the nanosecond transient absorption matrix (1 mJ pump) to a sequential model with three components.

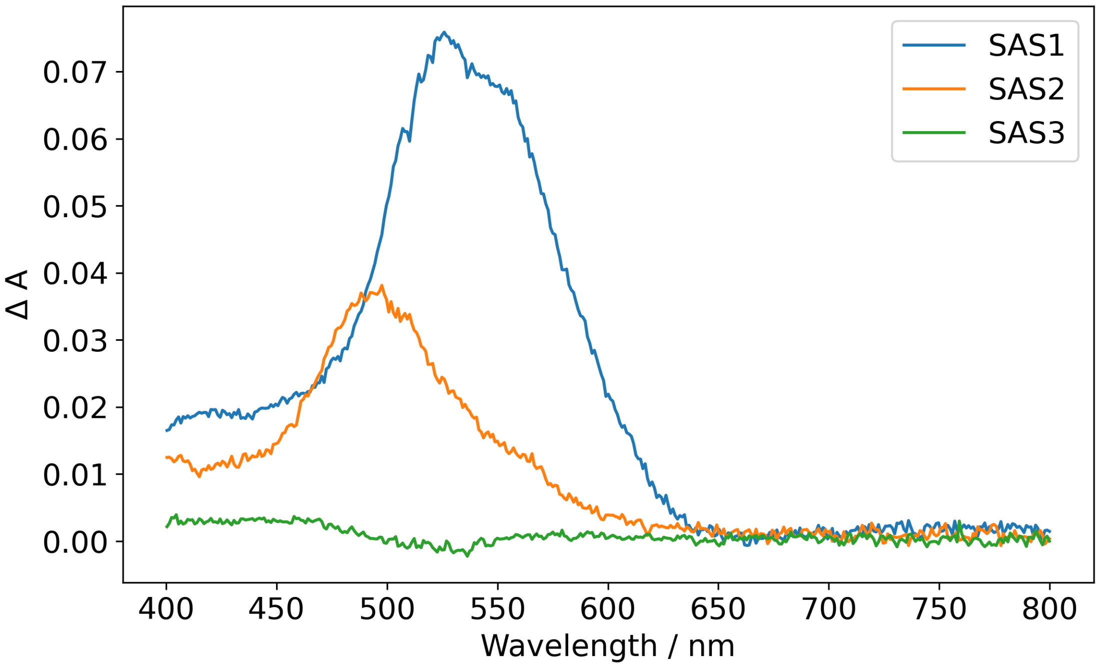

Figure S11. The species-associated spectra of ClC_{16} in DCM obtained from globally fitting the nanosecond transient absorption matrix (500 mJ pump) to a sequential model with three components.

Table S1. Lifetimes obtained from globally fitting the nanosecond transient absorption experiments using a three-step sequential model. The solvent used is DCM unless noted otherwise. The left column indicates the excitation pulse power used, and whether oxygen was present in the solution.

	τ_1(ns)	τ_2(μs)	τ_3(ms)
**C_8C_8**			
500 μJ	32	44	1.08
1 mJ	29	21	0.70
2 mJ	32	12	0.39
**C_8C_{16}**			
500 μJ	30	40	0.95
1 mJ	30	21	0.62
2 mJ	26	13	0.41
**$C_{16}C_{16}$**			
500 μJ	27	25	0.73
1 mJ	26	19	0.67
2 mJ	28	14	0.49
2 mJ, O_2	30	2	4.33
1 mJ, Dioxane	43	9	4.7
**ClC_{16}**			
500 μJ	124	9	7.6

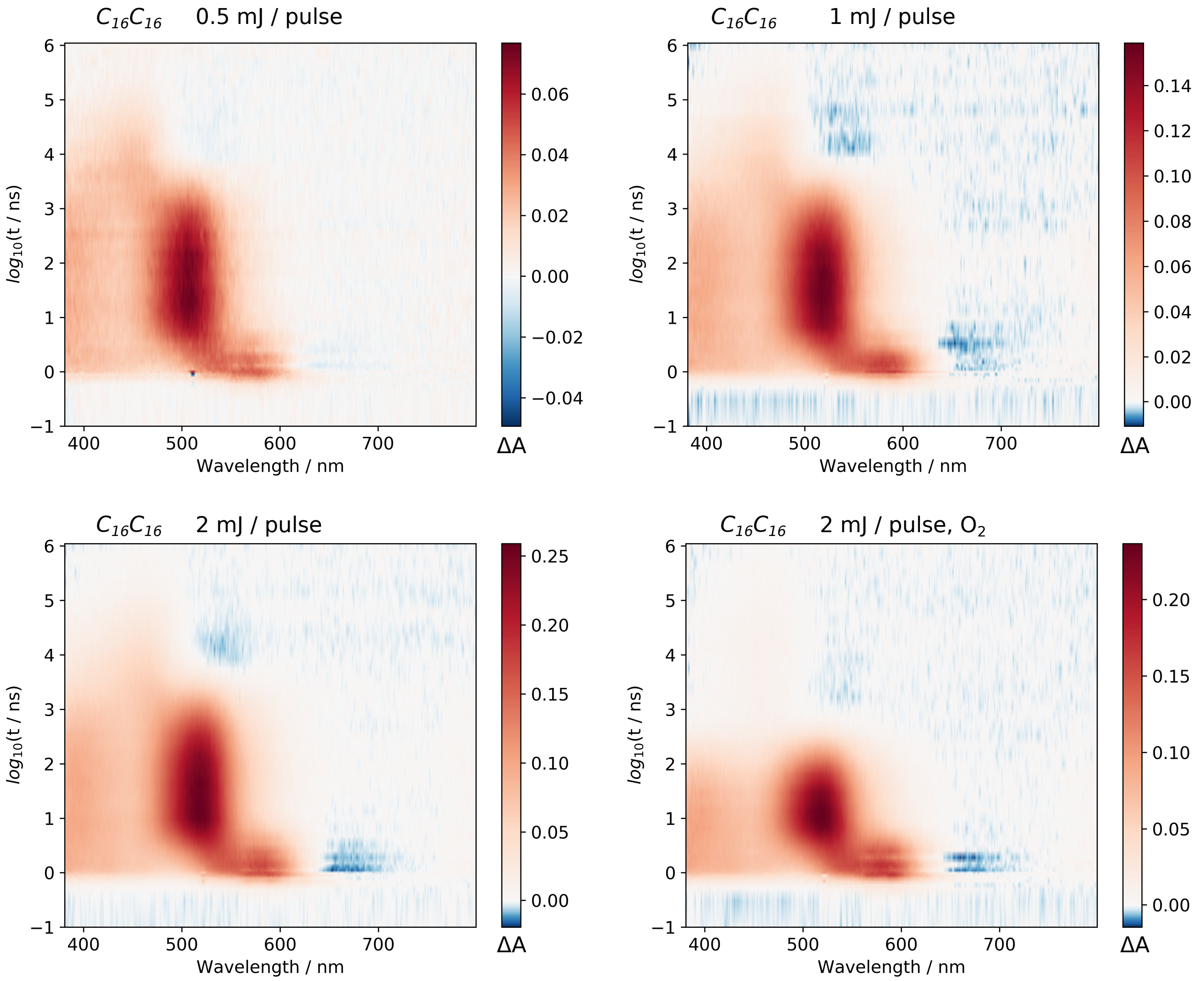

Figure S12. ns time-resolved transient matrix of $C_{16}C_{16}$ measured at different excitation powers and in the presence of oxygen.

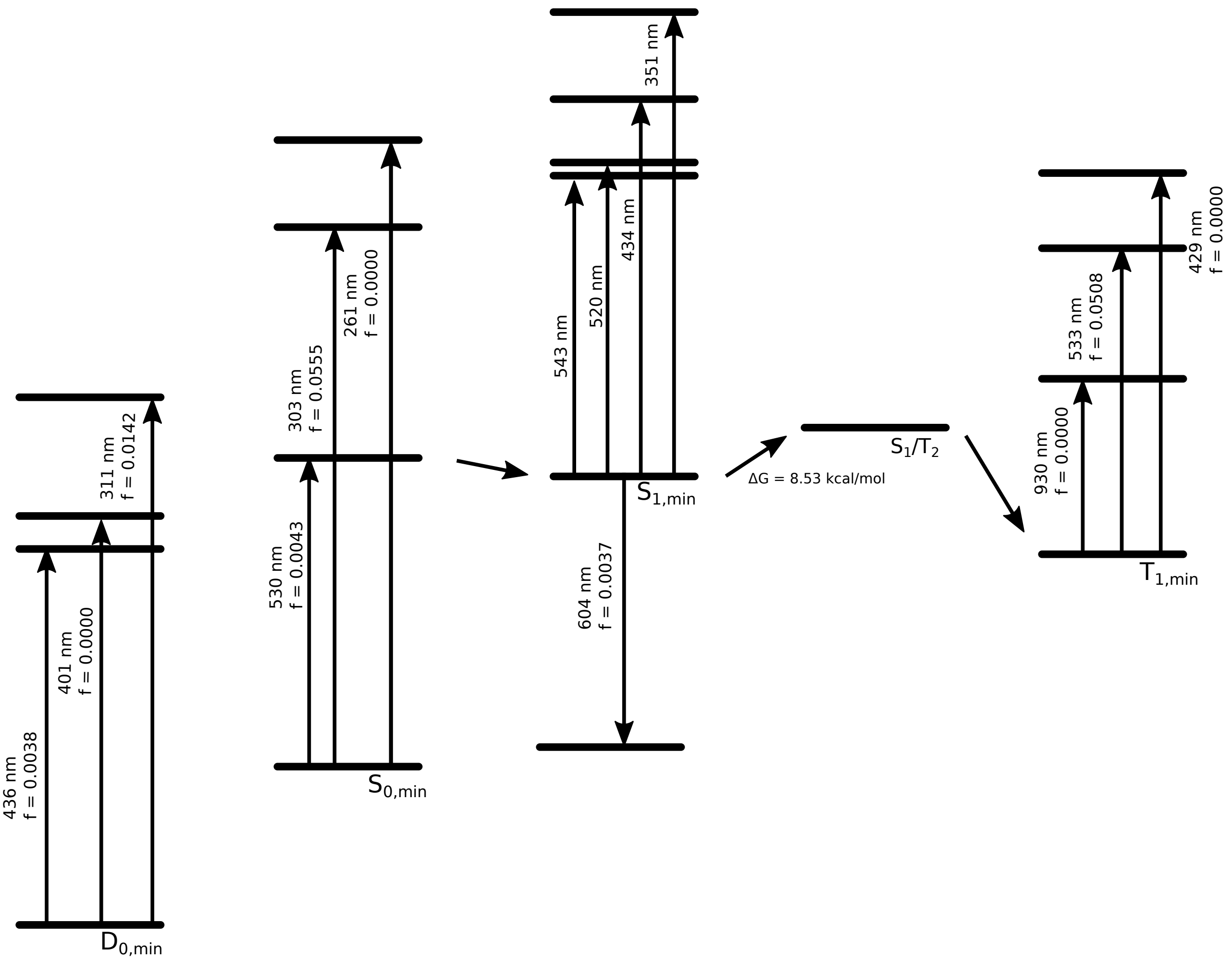

Figure S13. Energy diagram of C_1C_1 calculated at the ωB97XD/cc-pVTZ level of theory. The D_0 state refers to the anion.

Table S2. Energies in eV relative to the S_0 minimum computed at the ωB97XD/cc-pVTZ level of theory at the different minima and at the S_1/T_2 crossing (computed at the CASSCF/ANO-RCC-VDZP level) for ClC_1

	$\mathbf{S_{0,min}}$	$\mathbf{T_{1,min}}$	$\mathbf{S_{1,min}}$	$\mathbf{S_1/T_2}$
S_0	0.00	0.14	0.15	0.36
T_1	1.73	1.61	1.61	2.00
S_1	2.34	2.20	2.20	2.57
T_2	2.57	2.97	2.97	2.48
S_2	4.09	4.48	4.48	4.21

Table S3. Energies in eV relative to the S_0 minimum computed at the MS-CASPT2(14,10) level of theory at the different minima for C_1C_1

	$\mathbf{S_{0,min}}$	$\mathbf{T_{1,min}}$	$\mathbf{S_{1,min}}$	$\mathbf{S_1/T_2}$
S_0	0.00	0.17	0.19	0.35
T_1	1.62	1.55	1.55	1.90
S_1	2.18	2.09	2.09	2.45
T_2	3.40	3.95	3.97	3.31
S_2	4.04	4.23	4.46	4.71

Table S4. Computed transition energies, oscillator strengths, and initial anisotropies (r_0) relative to the $S_1 \leftarrow S_0$ transition of C_1C_1 calculated at the MS-CASPT2 level of theory at the S_1 minimum.

Transition	Wavelength / nm	Osc. strength	r_0
$S_1 \rightarrow S_0$	646	0.009	0.4
$S_2 \leftarrow S_1$	589	0.010	0.4
$S_3 \leftarrow S_1$	574	0.000	–
$S_4 \leftarrow S_1$	507	0.000	–
$S_5 \leftarrow S_1$	479	0.050	-0.2
$S_6 \leftarrow S_1$	320	0.020	-0.2

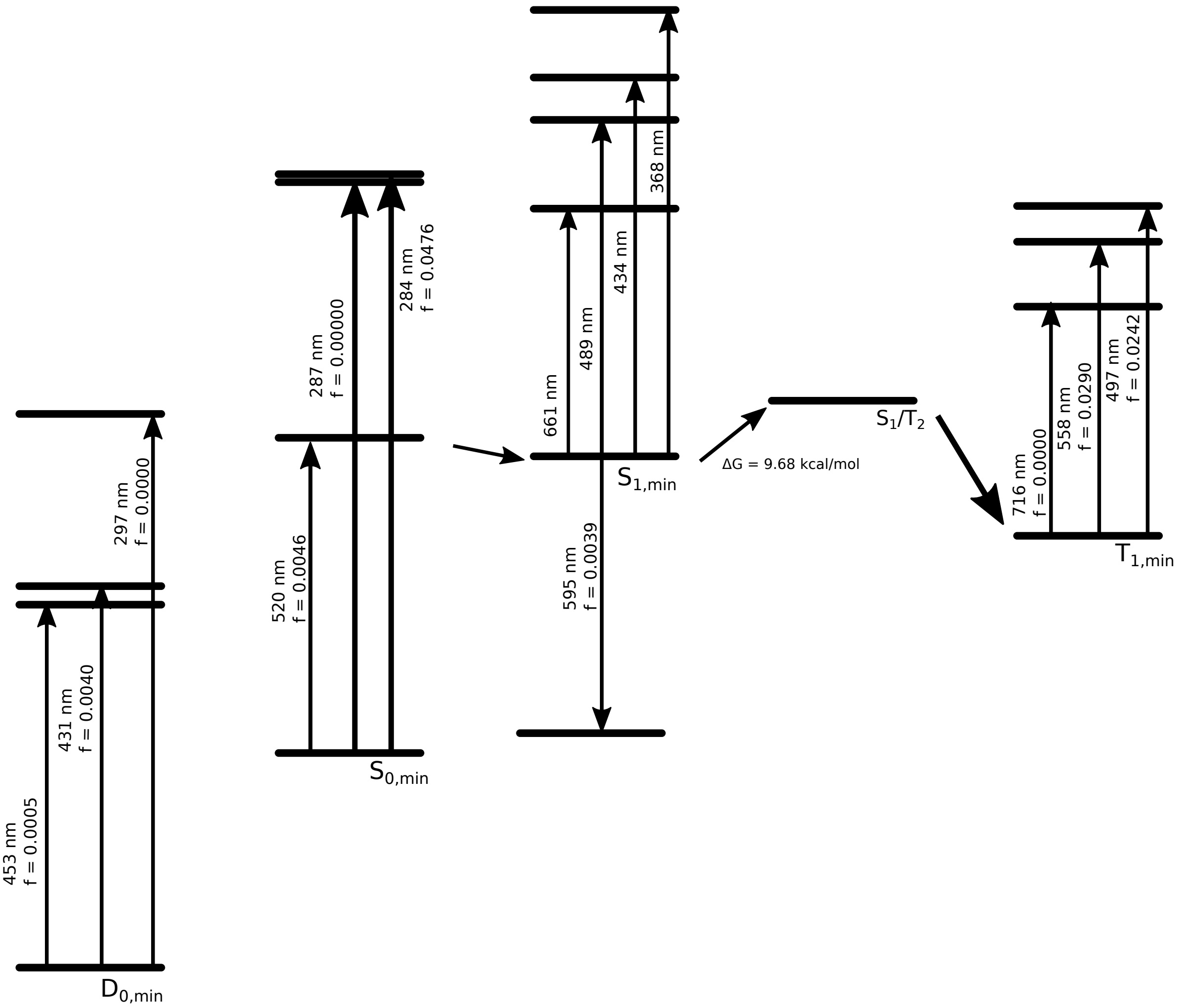

Figure S14. Energy diagram of ClC_1 calculated at the ωB97XD/cc-pVTZ level of theory. The D_0 state refers to the anion.

Table S5. Energies in eV relative to the S_0 minimum computed at the ωB97XD/cc-pVTZ level of theory at the different minima and at the S_1/T_2 crossing (computed at the CASSCF/ANO-RCC-VDZP level) for ClC_1

	$\mathbf{S_{0,min}}$	$\mathbf{T_{1,min}}$	$\mathbf{S_{1,min}}$	$\mathbf{S_1/T_2}$
S_0	0.00	0.14	0.15	0.55
T_1	1.77	1.64	1.64	2.07
S_1	2.38	2.24	2.24	2.66
T_2	3.00	3.37	3.37	2.89
S_2	4.31	4.14	4.14	4.56

Table S6. Energies in eV relative to the S_0 minimum computed at the MS-CASPT2(14,10) level of theory at the different minima for ClC_1

	$\mathbf{S_{0,min}}$	$\mathbf{T_{1,min}}$	$\mathbf{S_{1,min}}$	$\mathbf{S_1/T_2}$
S_0	0.00	0.23	0.25	0.50
T_1	1.66	1.58	1.58	1.97
S_1	2.22	2.16	2.16	2.48
T_2	4.14	4.61	4.61	3.48
S_2	4.32	3.87	3.85	4.76

Table S7. Computed transition energies, oscillator strengths, and initial anisotropies (r_0) relative to the $S_1 \leftarrow S_0$ transition of ClC_1 calculated at the M S-CASPT2(14,10) level of theory.

Transition	Wavelength / nm	Osc. strength	r_0
$S_1 \rightarrow S_0$	643	0.010	0.4
$S_2 \leftarrow S_1$	741	0.000	-0.2
$S_3 \leftarrow S_1$	605	0.010	0.4
$S_4 \leftarrow S_1$	464	0.073	-0.2
$S_5 \leftarrow S_1$	365	0.013	-0.2
$S_6 \leftarrow S_1$	342	0.000	0.4

Table S8. Geometric parameters that describe the s-tetrazine core of C_1C_1 at different energy minima

C_1C_1	N1-N2	N4-N5	N2-C3	C3-N4	N5-C6	C6-N1	C3-O	COC
S_0	1.304	1.304	1.331	1.331	1.331	1.331	1.324	117.5
S_1	1.301	1.301	1.325	1.326	1.325	1.326	1.315	116.9
S_1/T_2	1.343	1.342	1.281	1.39	1.281	1.39	1.309	118.5
$T1$	1.303	1.303	1.325	1.326	1.325	1.326	1.316	116.9

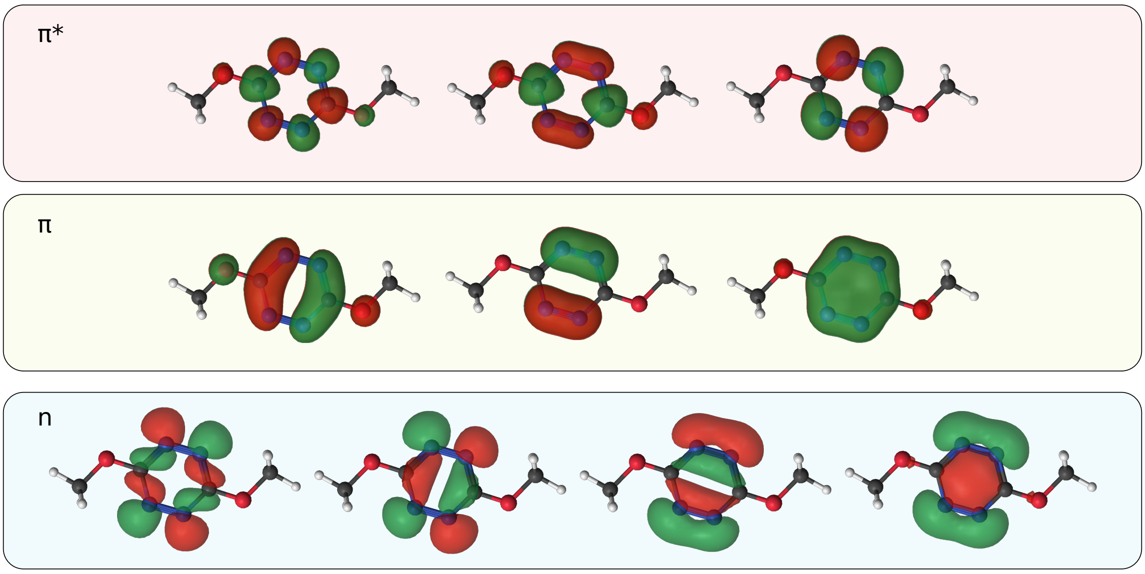

Figure S15. The active space of C_1C_1 used in MS-CASPT2(14,10) consisting of 4 n, 3 π, and 3 π^* orbitals.

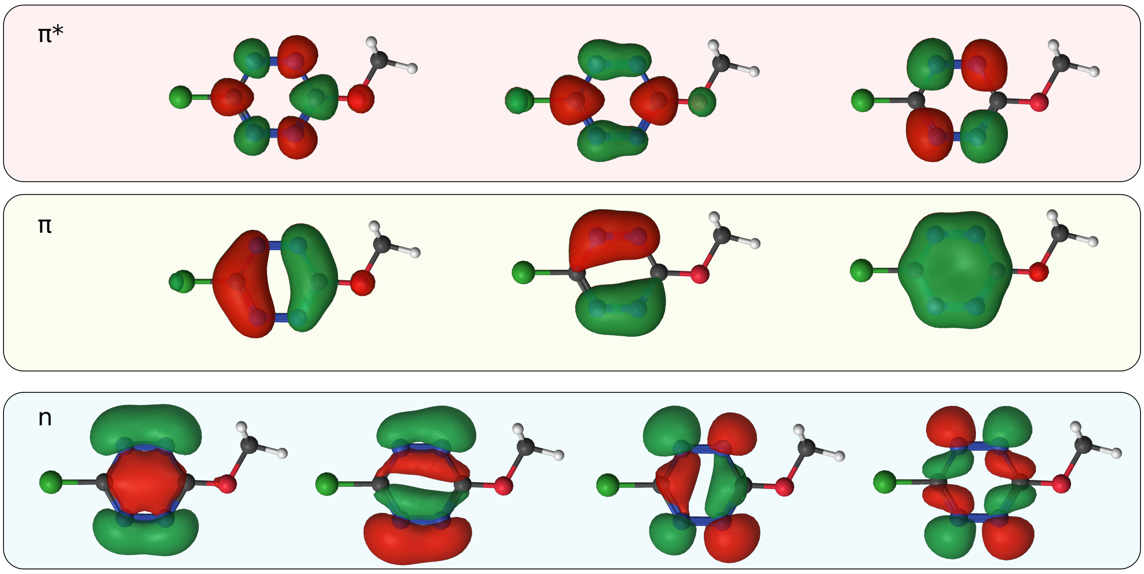

Figure S16. The active space of ClC_1 used in MS-CASPT2(14,10) consisting of 4 n, 3 π, and 3 π^* orbitals.

Table S9. Geometric parameters that describe the s-tetrazine core of ClC_1 at different energy minima

ClC_1	N1-N2	N4-N5	N2-C3	C3-N4	N5-C6	C6-N1	C6-O	C3-Cl	COC
S_0	1.295	1.314	1.335	1.316	1.327	1.343	1.316	1.718	117.7
S_1	1.295	1.308	1.318	1.318	1.326	1.332	1.307	1.706	117.2
S_1/T_2	1.335	1.344	1.284	1.373	1.283	1.381	1.307	1.71	118.4
T_1	1.294	1.313	1.32	1.316	1.324	1.333	1.308	1.707	117.2

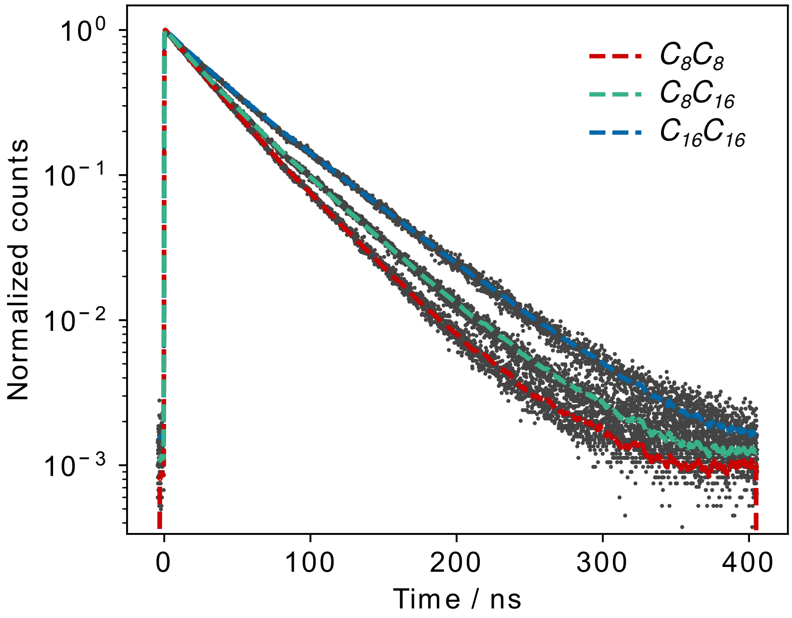

Figure S17. Normalized TCSPC decays of the different neat fluorescent liquids

Table S10. The lifetime of the fluorescent liquids in DCM, measured using TCSPC

	$\mathbf{A_1}$	$\mathbf{A_2}$	$\boldsymbol{\tau_1}$**/ns**	$\boldsymbol{\tau_2}$**/ns**
C_8C_8	0.11	0.89	11.3	35
C_8C_{16}	0.17	0.83	12	37
$C_{16}C_{16}$	0.08	0.92	13	38

Table S11. Fluorescence lifetimes of the neat liquids measured via TCSPC. The normalized emission intensity is fitted with the biexponential $A_1e^{-\frac{t}{\tau_1}} + A_2e^{-\frac{t}{\tau_2}}$.

	$\mathbf{A_1}$	$\mathbf{A_2}$	$\boldsymbol{\tau_1}$**/ns**	$\boldsymbol{\tau_2}$**/ns**
C_8C_8	0.07	0.93	16	39
C_8C_{16}	0.2	0.8	19	46
$C_{16}C_{16}$	0.17	0.83	20	54
ClC_8^a	0.07	0.97	1.70	4.72
ClC_{16}	0.17	0.83	16	49

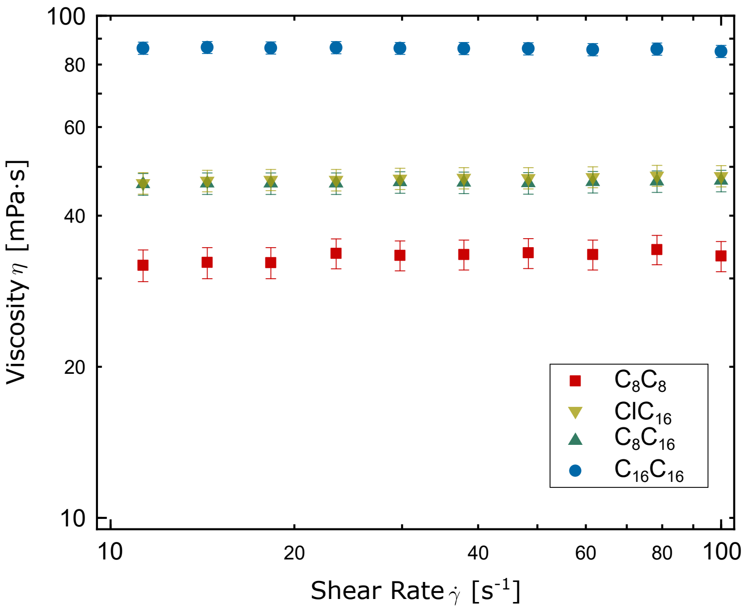

Figure S18. Viscosity η of the four fluorescent liquid derivatives as a function of the shear rate $\dot{\gamma}$. No shear thickening behavior is observed in the studied range of shear rate.
